# DPP7 promotes fatty acid β-oxidation in tumor-associated macrophages and determines immunosuppressive microenvironment in colorectal cancer

**DOI:** 10.7150/ijbs.117909

**Published:** 2025-10-01

**Authors:** Jiang Chang, Yuxu Niu, Shizhao Zhou, Weiying Zhu, Ziqi Zhang, Haoran Xiu, Ke Shang, Qingyang Feng, Ye Wei

**Affiliations:** 1 Department of General Surgery, Huadong Hospital Affiliated to Fudan University, Shanghai, China.; 2 Department of Thoracic Surgery, Shanghai East Hospital, School of Medicine, Tongji University, Shanghai, China.; 3 Colorectal Surgery Department, Zhongshan Hospital, Fudan University, Shanghai, China.; 4 Shanghai Engineering Research Center of Colorectal Cancer Minimally Invasive Technology, Shanghai, China.

**Keywords:** cancer immunotherapy, tumor-associated macrophage, fatty acid oxidation, dipeptidyl peptidase VII

## Abstract

**Background**: Tumor-associated macrophages (TAMs) are pivotal mediators of the immunosuppressive tumor immune microenvironment (TIME) in colorectal cancer (CRC). However, genes of TAMs that potentiate immunotherapy remain to be explored.

**Methods**: Single-cell RNA sequencing (scRNA-seq) data were analyzed to identify TAM molecular signatures, which were validated in patient cohorts from Huadong Hospital and TCGA to explore their clinical significance. Multidimensional characterization of CRC TIME and Dipeptidyl peptidase VII (DPP7)-positive TAMs functional state was achieved through cytometry by time-of-flight, multiplex immunofluorescence, in vitro and in vivo experiments. Mechanistic investigations integrating RNA-seq, Liquid Chromatography-Tandem Mass Spectrometry (LC-MS/MS)-based proteomics, and targeted lipid metabolomics have revealed the reprogramming of key metabolic pathways. Finally, the therapeutic potential of DPP7, which targets the enhancement of anti-PD-1 immunotherapy efficacy, was demonstrated.

**Results**: DPP7 was identified as the key gene in TAMs, and DPP7^+^TAMs correlated with metastasis and worse overall survival in multiple clinical cohorts. Functional characterization demonstrated that DPP7^+^TAMs drove the immunosuppressive TIME and promoted the exhaustion of CD8^+^T cells, thus exhibiting M2-polarized features. Mechanistically, DPP7 reduced ubiquitination-induced degradation of Carnitine Palmitoyltransferase 1A (CPT1A) by binding to CPT1A in a mutually exclusive manner with TRIM25, thus enhancing fatty acid oxidation (FAO) in TAMs. This metabolic reprogramming consumes lipids (including triglycerides and free fatty acids), elevates adenosine triphosphate (ATP) generation, and induces an immunosuppressive phenotype. In vivo, DPP7 knockdown in bone marrow-derived macrophages (BMDMs) synergized with anti-PD-1 therapy, achieving significant suppression of subcutaneous xenograft tumor growth and liver metastatic burden by reversing the immunosuppressive TIME.

**Conclusions**: DPP7 is mainly expressed in TAMs and DPP7^+^TAMs are strongly associated with adverse prognosis in CRC. Mechanistically, DPP7 enhances FAO to promote the M2-polarized phenotype in TAMs, leading to an immunosuppressive TIME. Targeting DPP7^+^TAMs may potentiate the efficacy of immunotherapy for CRC.

## 1. Introduction

Emerging from an expanded understanding of cancer biology and immunology, immune checkpoint inhibitor (ICI) therapy, represented by anti-PD-1/PD-L1 agents, has revolutionized solid tumor management and established itself as a cornerstone of oncological innovation in recent years^1^. However, this paradigm shift reveals striking limitations in colorectal cancer (CRC), the third most prevalent malignancy globally^2^. Current evidence indicates durable clinical responses to ICIs exclusively within the mismatch repair-deficient/microsatellite instability-high (dMMR/MSI-H) subgroup, representing only 15% of CRC cases^3^. Notably, even within the dMMR/MSI-H population, up to 50% of metastatic patients develop acquired resistance, culminating in therapeutic failure and disease recurrence^4^-^6^. The predominant "cold tumor" phenotype of CRC, marked by exhausted tumor-infiltrating lymphocytes and sophisticated immune evasion mechanisms, likely underlies this therapeutic inadequacy^7^, ^8^. Elucidation of these problems requires urgent investigation to optimize the immunotherapeutic strategies.

The therapeutic efficacy of ICIs fundamentally depends on the dynamic equilibrium between immunostimulatory and immunosuppressive cellular networks within the tumor immune microenvironment (TIME)^9^. As the predominant immune population, tumor-associated macrophages (TAMs) exhibit a functional dichotomy: classically activated M1 macrophages exert tumoricidal activity, whereas alternatively activated M2 macrophages propagate oncogenesis^10^. Crucially, M2-polarized TAMs orchestrate an immunosuppressive tumor microenvironment, thus facilitating neoplastic invasion, metastatic dissemination, and therapeutic resistance across multiple cancer types^11^-^13^. Research has revealed profound metabolic reprogramming in TAMs, with M2 subtypes demonstrating upregulated fatty acid oxidation (FAO), augmented oxidative phosphorylation, and accelerated tricarboxylic acid cycle (TCA)^14^. This metabolic reprogramming actively sustains their tumor-promoting phenotype^15^, ^16^. Given the remarkable plasticity of macrophage polarization, targeting pivotal metabolic regulators is a strategic imperative to subvert the suppressive TIME and potentiate ICI responsiveness in CRC.

Dipeptidyl peptidase VII (DPP7, also designated DPP2), a serine protease within the dipeptidyl peptidase family, catalyzes the cleavage of N-terminal dipeptides from proline-containing substrates and plays a conserved role in post-translational modifications^17^. This enzyme exhibits selective expression in quiescent lymphocytes, where its inhibition preferentially induces apoptosis in resting rather than antigen-activated lymphocyte populations, implicating DPP7 as a novel modulator of immune homeostasis^18^, ^19^. Clinically, elevated DPP7 expression correlates with worse survival prognosis in multiple myeloma and chronic lymphocytic leukemia^20^, ^21^. In CRC, emerging evidence also posits DPP7 as a prognostic biomarker, with its overexpression being strongly associated with reduced survival^22^, ^23^. Despite these clinical correlations, the cellular expression and mechanistic contributions of DPP7 to CRC pathogenesis, particularly its interplay with TIME, remain enigmatic, warranting systematic investigation.

In this study, single-cell RNA sequencing (scRNA-seq) was analyzed to identify DPP7, which was mainly expressed in TAMs in CRC patients. Through integrated clinical cohorts, multi-omics data, in vitro and in vivo experiments, we systematically demonstrated that DPP7 induced M2-like polarization by promoting FAO, thereby sculpting an immunosuppressive TIME. Mechanistically, DPP7 reduced ubiquitination-induced degradation of Carnitine Palmitoyltransferase 1A (CPT1A) by binding to CPT1A in a mutually exclusive manner with TRIM25 in TAMs. In vivo, DPP7 knockdown in bone marrow-derived macrophages (BMDMs) synergized with anti-PD-1 therapy, achieving significant suppression of subcutaneous xenograft tumor growth and liver metastatic burden by reversing the immunosuppressive TIME. Collectively, we recognized DPP7 as a central regulatory node governing TAM fatty acid metabolic reprogramming through CPT1A for the first time, revealing its potential as a novel therapeutic target to synergistically potentiate immunotherapy in CRC.

## 2. Materials and Methods

### 2.1 Patients and specimens

We conducted a retrospective analysis of 282 paired tumor and normal specimens from patients with CRC who underwent radical surgical resection at Huadong Hospital, Fudan University (Shanghai, China) between January 2017 and December 2018. The inclusion criteria were as follows: (a) age between 18 and 80 years and (b) pathological diagnosis of CRC. The exclusion criteria were: (a) multiple primary tumors or a history of other malignancies; (b) preoperative chemotherapy or radiotherapy; (c) incomplete clinicopathological and follow-up data, or unavailable surgical specimens; and (d) hereditary colorectal cancer (including familial adenomatous polyposis, Lynch syndrome). Baseline data for the Huadong cohort were summarized in Supplementary Table 1. Overall survival (OS) was calculated from the date of surgical intervention to patient death. Additionally, 8 freshly resected CRC specimens were acquired for cytometry by time-of-flight (CyTOF) analysis. All participants provided written informed consent and completed postoperative surveillance, with comprehensive clinicopathological parameters documented in the hospital's electronic medical record (EMR) system. Ethical approval for this study was obtained from the Clinical Research Ethics Committee of the Huadong Hospital, Fudan University (Approval ID: 20240172).

The colorectal carcinoma transcriptomic profiles and associated clinical metadata from The Cancer Genome Atlas (TCGA-COAD and TCGA-READ) were systematically downloaded from Genomic Data Commons (GDC, https://portal.gdc.cancer.gov/). For all RNA-seq data in this study, the “DESeq2” package in R software was used to normalize and calculate the differentially expressed genes. “ClusterProfiler” package was used to conduct Gene Ontology (GO) and Kyoto Encyclopedia of Genes and Genomes (KEGG) enrichment, and “GSVA” package was used to conduct Reactome pathway enrichment.

### 2.2 Single-cell RNA sequencing analyses

ScRNA-seq data from the public CRC patient cohort (GSE178341) were downloaded from the Gene Expression Omnibus (GEO) database, which included 26617 cells after quality control. Another CRC scRNA-seq dataset (PRJNA748525) was used for the validation. Quality control procedures involved exclusion of doublets (500 < nFeature_RNA < 5000 and 400 < nCount_RNA < 25000) and cells with > 30% mitochondrial gene content. After normalization and log-transformation of the transcriptomic data, 2,000 highly variable genes were identified for subsequent principal component analysis (PCA), based on the average expression levels and dispersion of the genes. The first 20 principal components (p<0.05) were used for t-distributed stochastic neighbor embedding (t-SNE) analysis to identify distinct single-cell subclusters. Cell cluster annotation was performed, and myeloid cells were further divided based on a comprehensive assessment of CellMarker 2.0 (Supplementary Fig.1A-E). All analyses were performed using the 'Seurat' package in R software. To investigate the intricate interactions between different cell clusters, a cell-cell communication network was constructed using the “CellChat” package.

### 2.3 Immunohistochemistry (IHC) and immunofluorescence (IF) assay

IHC and IF staining of formalin-fixed, paraffin-embedded (FFPE) tissues were performed as previously described^24^. In summary, tissue sections were incubated with the appropriate primary antibodies overnight at 4 °C, followed by the application of DAB staining. The expressions of DPP7, CD68 and PD-L1 were quantified by integrating the IHC staining intensity (scored as 0 for negative, 1 for weak, 2 for moderate, and 3 for strong) with the percentage of positive cells (scored as 0 for 0-5%, 1 for 6-25%, 2 for 26-75%, and 3 for >75%), yielding a composite score ranging from 0 to 9. DPP7^+^TAMs were defined as DPP7^+^CD68^+^ double-positive cells by IF, in which sections were similarly incubated overnight at 4 °C with primary antibodies and subsequently treated with the corresponding secondary antibodies at room temperature. The slides were then mounted using an Antifade Mounting Solution containing DAPI and subjected to further analysis. Two pathologists, blinded to the clinical and pathological data, independently quantified the slides in five randomly selected high-power fields. In cases where the difference between the pathologist counts exceeded 10%, a recount was performed.

Patients were categorized into high and low groups based on the cut-off value calculated using X-tile software (version 3.6.1). For IHC results, the optimal cut-off proportion of DPP7 for OS prediction in the Huadong cohort was determined to be 37%, where patients in the top 37% of DPP7 expression were categorized as the DPP7 high group, with the remaining 63% classified as the low group. Similarly, CD68 exhibited an optimal survival predictive cutoff at the 45th percentile. In the TCGA validation cohort, consistent grouping cutoffs (37% for DPP7 and 45% for CD68) were applied to define high- and low-expression groups. For IF quantification, the infiltration density of DPP7^+^TAMs was calculated, and the optimal prognostic cut-off proportion was determined to be 49%.

The following primary antibodies were used: DPP7, 1:400, 19018-1-AP, Proteintech; CD68, 1:1500, 66231-2-Ig, Proteintech; PD-L1, 1:500, 28076-1-AP, Proteintech.

### 2.4 Cytometry by time-of-flight (CyTOF)

Freshly excised tumor specimens from eight CRC patients were processed into single-cell suspensions using the Tumor Dissociation Kit (130-096-730, Miltenyi Biotec) and 70μm filtration. After centrifugation and lysis of erythrocytes, cells were incubated with a panel of 41 metal-conjugated antibodies. Signal acquisition was conducted using the Helios3 CyTOF system (PLTTech, Hangzhou, China). The data were normalized and analyzed using the Cytobank platform (https://www.cytobank.org/). To identify distinct cell populations, unsupervised clustering and t-SNE dimensionality reduction were performed based on the expression profiles of the markers, utilizing the “cytofkit” package in R. Detailed antibody information was summarized in Supplementary Table 5.

### 2.5 Multiplex immunofluorescence assay (mIF)

Briefly, 20 CRC sections were deparaffinized and endogenous peroxidase was blocked according to the manufacturer's instructions as previously reported^25^. After blocking with 3% BSA in TBST for 30 min, the sections were incubated with the antibody DPP7 (1:400, 19018-1-AP, Proteintech) for 30 min, which was detected using the corresponding secondary antibody tagged with HRP for further visualization. Subsequently, antigens were retrieved again and all samples were stained sequentially with CD68 (1:1500, 66231-2-Ig, Proteintech), CD8 (1:200, ab101500, Abcam), PD-1 (1:500, ab237728, Abcam), and panCK (1:200, ab7753, Abcam). The slides were counterstained with DAPI for 10 min and coverslipped using an anti-fade mountant.

### 2.6 Cell lines

Commercially available human embryonic kidney cell line 293T (HEK293T), human monocytic-leukemia cell line THP-1, and murine colon cancer cell line MC38 were purchased from the Stem Cell Bank, Chinese Academy of Science (Shanghai, China). HEK293T cells were cultured in high-glucose Dulbecco's modified Eagle's medium (DMEM), whereas THP-1 and MC38 cells were maintained in Roswell Park Memorial Institute (PRMI) 1640 medium. Both media were supplemented with 10% fetal bovine serum (FBS), 1% antibiotics (100U/mL penicillin‒streptomycin) and incubated at 37 °C in a humidified incubator containing 5% CO2. THP-1-differented macrophages were induced by adding 100 ng/ml PMA (Sigma) for 48 h.

### 2.7 Mouse primary cells

Bone marrow-derived macrophages (BMDMs) were obtained as previous studies^26^. Briefly, to isolate BMDMs, the femurs, tibias, and iliac bones of C57BL/6J mice were washed with PBS. Red blood cells were removed by treatment with a red blood cell lysis buffer. Bone marrow cells were subsequently plated onto culture dishes in DMEM supplemented with 10% FBS, 20 ng/mL M-CSF (Sigma), and 100 U/mL penicillin-streptomycin for 7 days.

### 2.8 Co-culture assay

CD8^+^T cells were isolated from the spleens of wild-type C57BL/6 mice using a magnetic bead-based isolation kit (480035, BioLegend). Purified cells were quantified and plated in anti-CD3/CD28 pre-coated (2μg/mL, 100340 BioLegend, 102116 BioLegend) 96-well plates. The complete culture medium contained RPMI 1640 with 10% FBS, 100 U/mL IL-2, and standard supplements (2 mM glutamine, 50 μM β-mercaptoethanol, 1 mM pyruvate, non-essential amino acids, and 10 mM HEPES). After 24h of pre-activation, the cells were co-cultured with macrophages (1:1 ratio) for 48 h. The function of CD8^+^T cells was evaluated by flow cytometry.

### 2.9 Plasmids, short hairpin RNA (shRNA) construct and transduction

The full-length human DPP7 cDNA sequence was inserted into the lentiviral pCDH-G418-3xFlag plasmid. Full-length human CPT1A cDNA sequence was inserted into the lentiviral pCDH-Puro-GST and pCDH-Puro-Myc plasmids. The full-length human TRIM25 cDNA sequence was inserted into the lentiviral pCDH-G418-6xHis plasmid. ShRNAs targeting human DPP7, TRIM25, and mouse DPP7 were inserted into the LV3 (-puromycin) plasmid.

To construct stable cell lines overexpressing or knocking down DPP7, TRIM25, and CPT1A, lentiviral vectors were constructed based on the above plasmids and were designated accordingly. The cells were transduced with lentivirus and maintained in the presence of puromycin or G418.

For BMDMs, lentiviral supernatants were added to the culture medium for 12h. The medium was replaced for 48h, followed by using 1ug/ml puromycin for 3 days. See Supplementary Table 6 for the shRNA sequences.

### 2.10 RNA isolation and quantitative real-time PCR (qPCR)

Total RNA was isolated from the cells using TRIzol reagent (Invitrogen), followed by reverse transcription into cDNA using the PrimeScript RT Reagent Kit (Takara), in accordance with the manufacturer's protocols. For quantitative real-time PCR (qPCR), the reaction mixture, consisting of cDNA, primers, and SYBR Green Master Mix, was run on an ABI Prism 7500 Sequence Detection System (Applied Biosystems). GAPDH was used as an internal control. See Supplementary Table 7 for primer sequences.

### 2.11 Western blot (WB) assay and co-immunoprecipitation (Co-IP)

WB was performed by lysing the cultured cells in RIPA buffer containing protease inhibitors to extract proteins, which were then quantified using a BCA assay kit (Thermo, Fisher Scientific). Proteins were denatured by heating, combined with 5x loading buffer, and separated by SDS-PAGE before being transferred onto PVDF membranes (Millipore). Membranes were probed with specific primary antibodies and HRP-conjugated secondary antibodies. Detection was performed using enhanced chemiluminescence reagents and visualized using an ImageQuant™ LAS 4000 system. See Supplementary Table 8 for the antibodies used in WB.

For Co-IP, cell lysates were incubated with the specific antibodies overnight at 4 °C on a rotating platform. The mixture was then incubated at room temperature for 30 min with protein A/G beads (Thermo, Fisher Scientific) under gentle agitation. The beads were then washed five times with chilled lysis buffer and eluted by resuspension in SDS-PAGE loading buffer for further analysis by immunoblotting. In the ubiquitination assay, cells were transduced with the indicated plasmids and the resulting lysates were immunoprecipitated with anti-CPT1A antibodies. The immunoprecipitates were analyzed by western blotting using a primary antibody against ubiquitin. See Supplementary Table 8 for the antibodies used in the Co-IP.

### 2.12 Pull-down assay

Equal quantities (200 ng/sample) of His-tagged purified TRIM25 protein were mixed with 100 ng of GST or GST-fusion protein of CPT1A, along with glutathione agarose beads, and incubated for 2 h at 4 °C. Similarly, GST-tagged purified CPT1A protein was incubated with 100 ng of His-GFP or His-fusion protein of TRIM25. After incubation, the beads were subjected to four washes with a modified binding buffer and then proteins were eluted for WB, using the specified antibodies for detection.

### 2.13 Cell immunofluorescence staining and confocal microscopy

The cells were seeded into glass-bottom culture dishes and allowed to adhere for 24 h. After fixation with 4% paraformaldehyde (Beyotime) for 20 min, cell membranes were permeabilized with 0.3% Triton X-100 (Beyotime). Following a 1-hour blocking step with 5% goat serum, cells were incubated overnight at 4 °C with primary antibodies (DPP7, 1:100, PA5-54369, Invitrogen; CPT1A, 1ug/ml, ab128568, Abcam; TRIM25, 1:100, PA5-30640, Invitrogen) according to the manufacturer's recommendations. The following day, the cells were incubated with Alexa Fluor 488-labeled goat anti-mouse IgG (ab150113, Abcam) or Alexa Fluor 594-labeled goat anti-rabbit IgG (ab150080, Abcam) for 1 h. Nuclei were stained with DAPI (Beyotime), and images were acquired using a confocal fluorescence microscope (Leica).

### 2.14 Flow cytometry (FCM)

To generate a single-cell suspension, Accutase (Sigma) was used to dissociate macrophages derived from THP-1 cells, which were resuspended in PBS. For tumor tissues from mice, the samples were finely minced and incubated with collagenase IV (Sigma) and DNase I (Sigma) for 1-2 hours at 37 °C, after which they were filtered through a 70 μm nylon mesh to separate the cells. The resulting cells were stained with a fixable viability dye (Thermo, Fisher Scientific), followed by permeabilization using a Fixation and Permeabilization Solution (Thermo, Fisher Scientific). After washing three times with PBS, the cells were exposed to fluorochrome-conjugated antibodies at 4 °C in the dark for 30 min. Flow cytometric analysis was conducted using a BD FACSAria Flow Cytometer, and data analysis was performed using FlowJo software. See Supplementary Table 9 for the antibodies used in the FCM.

### 2.15 Oil red O Staining, free fatty acid assay, adenosine triphosphate (ATP) assay and fatty acid oxidation experiments

Cellular metabolites were analyzed following the manufacturer's protocols for the corresponding commercial assay kits, including the modified Oil Red O Staining Kit (Beyotime) for triglyceride, Amplex Red Free Fatty Acid Assay Kit (Beyotime) for free fatty acids, and ATP Assay Kit (Beyotime) for ATP.

Fatty acid oxidation experiments were performed as described^27^. Briefly, the cells were incubated overnight in a substrate-limited growth medium (XF DMEM medium (Agilent) with 0.5 mM Glucose (Agilent), 1 mM Glutamine (Agilent), 1%FBS, and 0.5 mM L-Carnitine (MCE) and then replaced with a substrate-limited assay medium (XF DMEM with 2.0 mM Glucose and 0.5 mM L-carnitine) and in response to 1 μM Oligomycin (Selleck Chemicals), 1 μM fluoro-carbonyl cyanide phenylhydrazone (FCCP) (Selleck Chemicals), and 1μM complex I/III inhibitors (Rotenone, Sigma, Antimycin A, Sigma). Data processing was performed using the manufacturer's proprietary Mito Stress Test Analysis Suite (Agilent).

### 2.16 Reagents

The following reagents used in the experiments: Etomoxir (HY-50202), cycloheximide (CHX; HY-12320), MG-132 (HY-13259), and chloroquine (HY-17589A) were all purchased from MedChemExpress (USA). Etomoxir, an inhibitor of CPT1A, was applied to inhibit CPT1A, thus suppressing FAO in vitro. Cycloheximide (CHX), a natural antibiotic produced by Streptomyces griseus, was used in this study to inhibit eukaryotic protein synthesis by targeting the 60S ribosomal subunit. MG132 is a widely used proteasome inhibitor. In this study, it was used to block the degradation of ubiquitin-tagged proteins through the ubiquitin-proteasome pathway. Chloroquine was used to effectively inhibit protein degradation via the autophagy-lysosome pathway.

### 2.17 Animal experiments

All animal experiments in this study were approved by the ethics committee of Fudan University [2024-HDYY-066] and designed according to previous studies^26^, ^28^. For subcutaneous xenograft tumor formation experiments, C57BL/6J mice (6-8 weeks) and BALB/c-nu mice (6-8 weeks) were randomly divided into different groups. A mixture of 200μl MC38 cells (1 × 10^6^) and BMDMs-shDPP7 (1 × 10^6^) or BMDMs-shNC (1 × 10^6^) was injected into the flanks of the mice. The tumor size was measured every 3 days and the volume was calculated according to the following formula: volume = 1/2 × (width^2^ × length). The mice were sacrificed after 18 days and the tumor volume and weight were measured. For the liver metastasis model, anesthesia was induced in mice using isoflurane, followed by laparotomy of the left abdomen. CRC cells (100ul, 1 × 10^5^) and BMDMs-shDPP7 (1 × 10^5^) or BMDMs-shNC (1 × 10^5^) were slowly injected into the spleens. Mice were humanely euthanized at the study endpoint, 17 days post-inoculation, for subsequent analysis and tissue collection. Macroscopic evaluation of metastatic liver tumors was performed by counting the visible tumors and measuring their largest dimensions.

For CD8^+^T cell depletion, mice were intraperitoneally injected with 200 μg anti-CD8 (BP0061, BioXcell) or control IgG 1 day before tumor injection and every 4 days thereafter.

For anti-PD-1 treatment, mice received intraperitoneal injections of 200µg of anti-mouse PD-1 antibody (BP0273, BioXCell) or an IgG isotype control on day 7 post tumor implantation for the first time, once every 3 days for a total of 4 doses.

### 2.18 Statistics and reproducibility

All experiments were performed at least three times. Statistical analyses were performed using the GraphPad Prism Software v.8.0. For continuous variables comparing two independent groups, the data distribution was first assessed using the Shapiro-Wilk normality test. Where normality assumptions were violated (p < 0.05), the Mann-Whitney U test was employed. For normally distributed variables, the homogeneity of variances was verified using Levene's test. If equal variances were confirmed (p ≥ 0.05), Student's t-test was applied; when variances were unequal (p < 0.05), Welch's t-test (also known as t'-test or unequal variances t-test) was utilized. Categorical variables were analyzed using Pearson's chi-square test, Yates' continuity correction and Fisher's exact test when appropriate. Significant P-values were indicated by asterisks in the figures as follows: * p < 0.05, ** p < 0.01, *** p < 0.001, and ns = non-significant. The survival curve was constructed using the log-rank test, and a p-value of <0.05 was considered statistically significant.

## 3. Results

### 3.1 Single-cell transcriptome sequencing analyses identify macrophage markers associated with CRC prognosis

**Figure 1:**
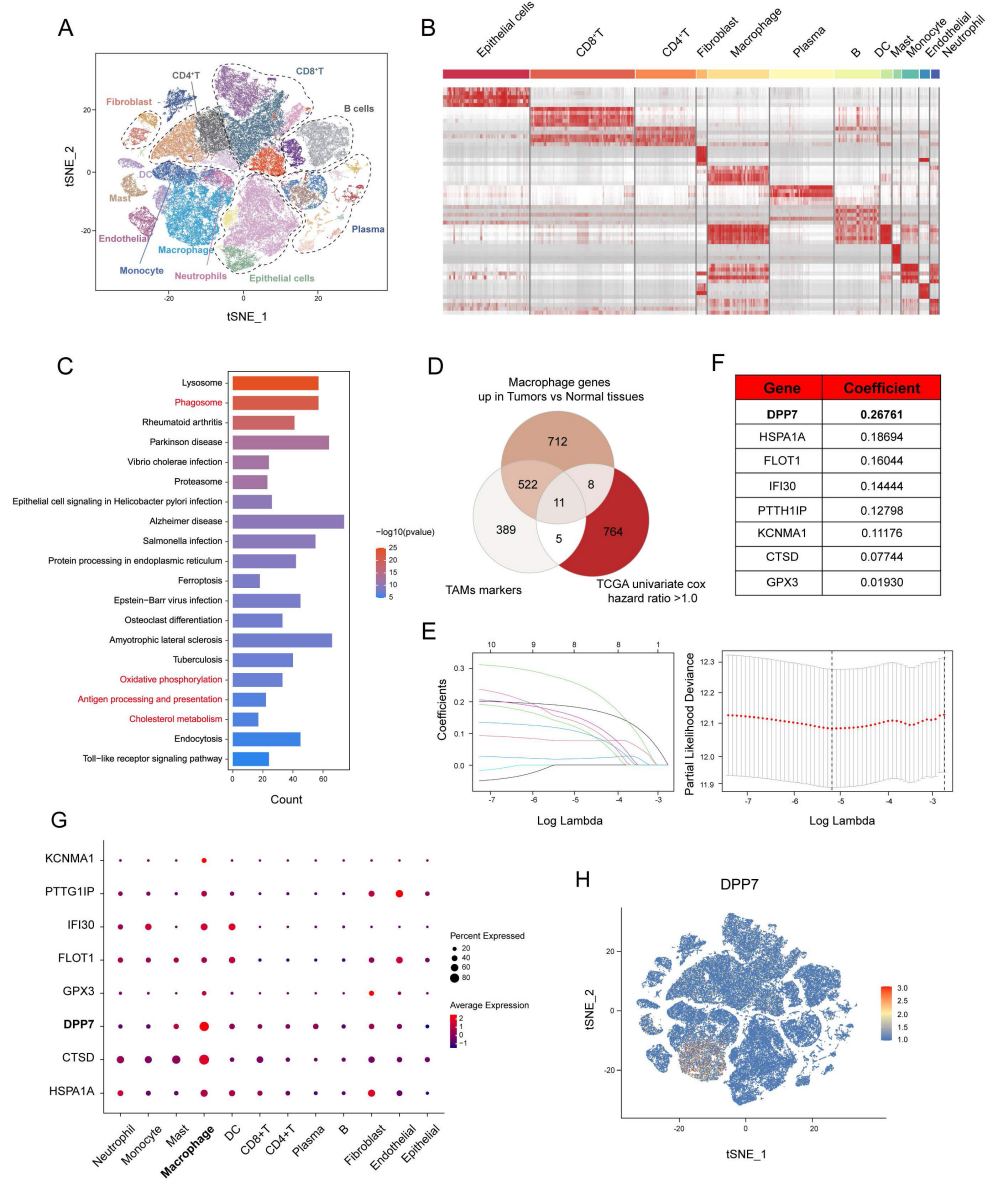
** Single-cell transcriptome sequencing analyses identify macrophage markers associated with colorectal cancer prognosis.** (A) t-SNE plot of scRNA-seq consisting of 64 colorectal cancer tissues and 36 normal tissues in the GSE178341 cohort. (B) Marker genes expression heatmap across scRNA-seq annotated subclusters. (C) KEGG pathway enrichment analysis of macrophage genes up-regulated in tumor tissues compared with normal tissues. (D-F) Identification of macrophage genes of CRC through intersection of three gene sets and Lasso analysis. (G) Dot plot demonstrating subcluster expression patterns of identified genes. (H) Feature plot of all single cells colored by the expression level of DPP7.

To delineate the transcriptomic profile of TAMs in CRC, we analyzed a public scRNA-seq dataset (GSE178341, 64 CRC, and 36 normal tissues). t-SNE dimensionality reduction of the top 20 principal components resolved 12 cell populations (Fig.1A) annotated via canonical markers (Supplementary Fig.1A-E), including epithelial cells, CD8^+^T cells, CD4^+^T cells, fibroblasts, macrophages, plasma cells, B cells, DC cells, mast cells, monocytes, endothelial cells, and neutrophils. The significantly distinct transcriptional profiles of these cell subclusters were shown (Fig.1B). KEGG pathway analysis of macrophages in tumor versus normal tissue identified differential enrichment in phagocytosis, metabolic regulation, and antigen presentation pathways (Fig.1C), which indicated obvious biological changes within TAMs. To prioritize functional targets, we selected three gene sets: (1) scRNA-seq-derived TAMs markers, (2) genes upregulated in macrophages in tumor versus normal tissue, and (3) worse prognosis-related genes (univariate Cox analysis hazard ratio>1.0) from TCGA cohort (TCGA-COAD+TCGA-READ; n=592) (Fig.1D). LASSO (Least absolute shrinkage and selection operator) regression with 1000-fold cross-validation refined this intersection into eight candidate genes: DPP7, HSPA1A, FLOT1, IFI30, PTTG1IP, KCNMA1, CTSD, and GPX3 (Fig.1E, F). DPP7 achieved the highest coefficient (Fig.1F), and was highly expressed in macrophages, moderately expressed on other myeloid cells, and showed lower expression on lymphoid cells and other cell types (Fig.1G, H), which was validated using another CRC scRNA-seq dataset (PRJNA748525, Supplementary Fig.2). Moreover, we observed that the expression level of DPP7 was significantly higher in CRC tissues compared to normal tissues (Supplementary Fig.1F, G). Overall, our scRNA-seq analyses reveal a panel of genes that may play a role in TAMs and affect the prognosis of CRC patients.

### 3.2 Infiltration of DPP7^+^TAMs is significantly correlated with prognosis in patients with CRC

**Figure 2:**
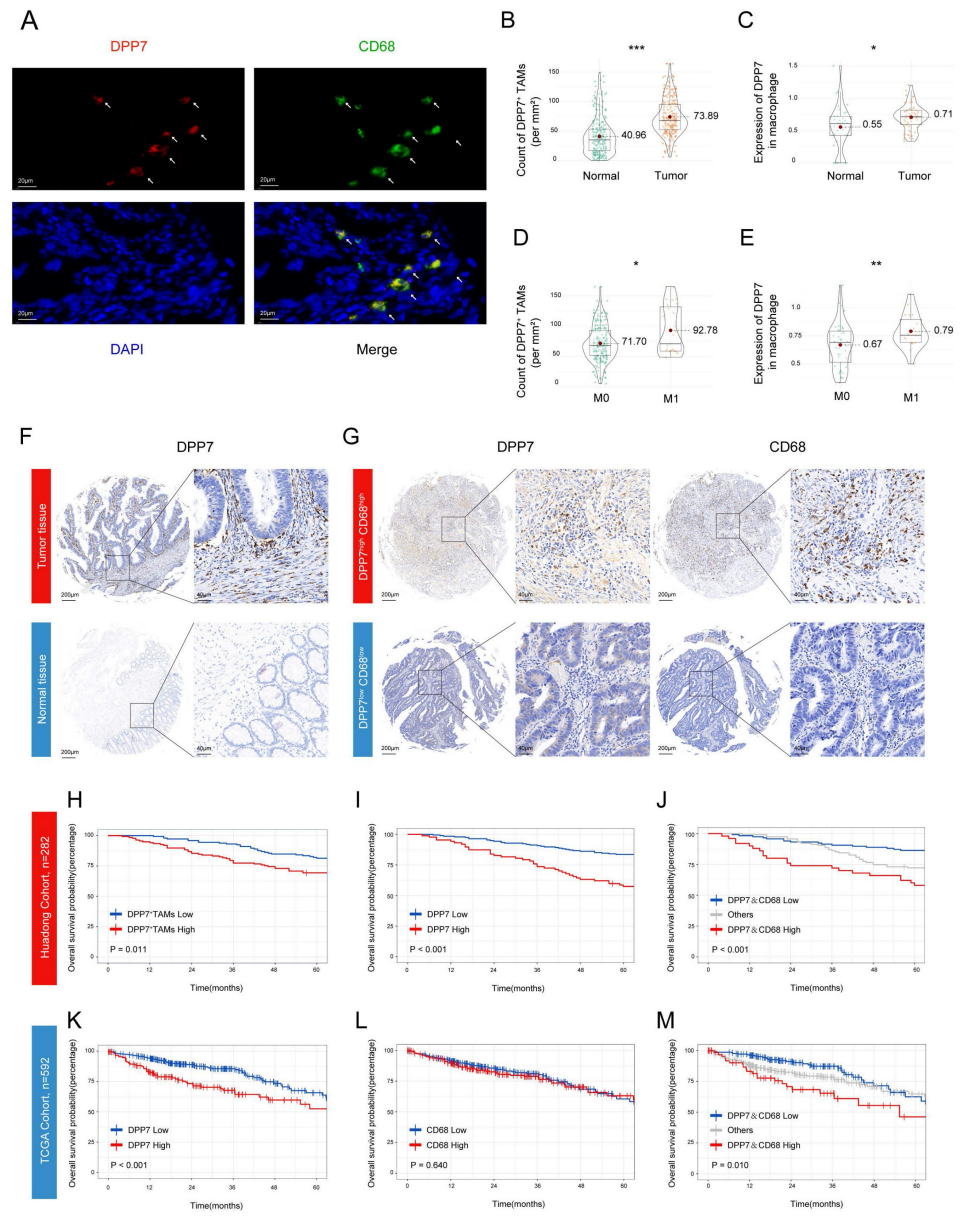
** Infiltration of DPP7^+^TAMs is significantly correlated with prognosis in patients with CRC.** (A) Representative images of IF co-staining of DPP7 (red) and CD68 (green) as DPP7^+^TAMs in CRC tissues from the Huadong hospital cohort. (B, D) The density of DPP7^+^TAMs in different groups based on IF co-staining. (C, E) The expression of DPP7 in macrophages in different groups based on scRNA-seq. (F) Representative IHC images of DPP7 in CRC tissues and normal tissues. (G) Representative IHC images of DPP7 and CD68 in CRC tissues. (H) OS curve for CRC patients with high and low DPP7^+^TAMs infiltration based on double-labeling IF staining in Huadong patient cohort (n=282). (I-J) OS curves for CRC patients with high and low expression of DPP7 and CD68 based on IHC staining in the Huadong hospital cohort (n=282). (K-M) OS curves for CRC patients with high and low expression of DPP7 and CD68 based on IHC staining in TCGA cohort (n=592). *p<0.05, **p<0.01, ***p<0.001, and ns = non-significant.

Next we investigated the clinical value of DPP7^+^TAMs. Through dual-labeled IF assays conducted on CRC specimens from the Huadong hospital cohort, we identified the colocalization of DPP7 with the macrophage marker CD68 in tumor tissues (Fig.2A, representative images of normal tissues see Supplementary Fig.3A), which was defined as DPP7^+^TAMs. Quantitative analysis demonstrated enhanced infiltration of DPP7^+^TAMs within tumor tissues compared to normal mucosa (Fig.2B), with preferential accumulation in patients with metastatic lesions (Fig.2D), which was corroborated by scRNA-seq analyses (Fig.2C, E). Higher preoperative serum carcinoembryonic antigen (CEA) levels and larger tumor sizes were also observed in the high DPP7^+^TAMs group (Supplementary Table 2). Furthermore, IHC validation confirmed both the upregulation of DPP7 in tumor and its positive correlation with CD68 expression (Fig.2F, G, Supplementary Fig.3B, C). Prognostic evaluation using the Kaplan-Meier method revealed significant clinical value. Patients with high DPP7^+^TAMs infiltration demonstrated markedly inferior survival compared to those with low infiltration in the Huadong cohort (Fig.2H). Mirroring these findings, higher DPP7 expression correlated with reduced OS (Fig.2I), whereas dual overexpression of CD68 and DPP7 showed the worst clinical outcome (Fig.2J), but CD68 alone did not (Supplementary Fig.3D). This prognostic paradigm was further validated in the TCGA cohort (Fig.2K, L, M). Multivariate Cox regression analysis confirmed that the density of DPP7^+^TAMs in CRC was an independent prognostic factor for OS (p = 0.037, Table 1). Taken together, our data demonstrate that a high intratumoral density of DPP7^+^TAMs serves as a prognostic indicator for adverse clinical outcomes.

### 3.3 DPP7^+^TAMs promote the formation of immunosuppressive TIME and the exhaustion of CD8^+^T cells in CRC

**Figure 3:**
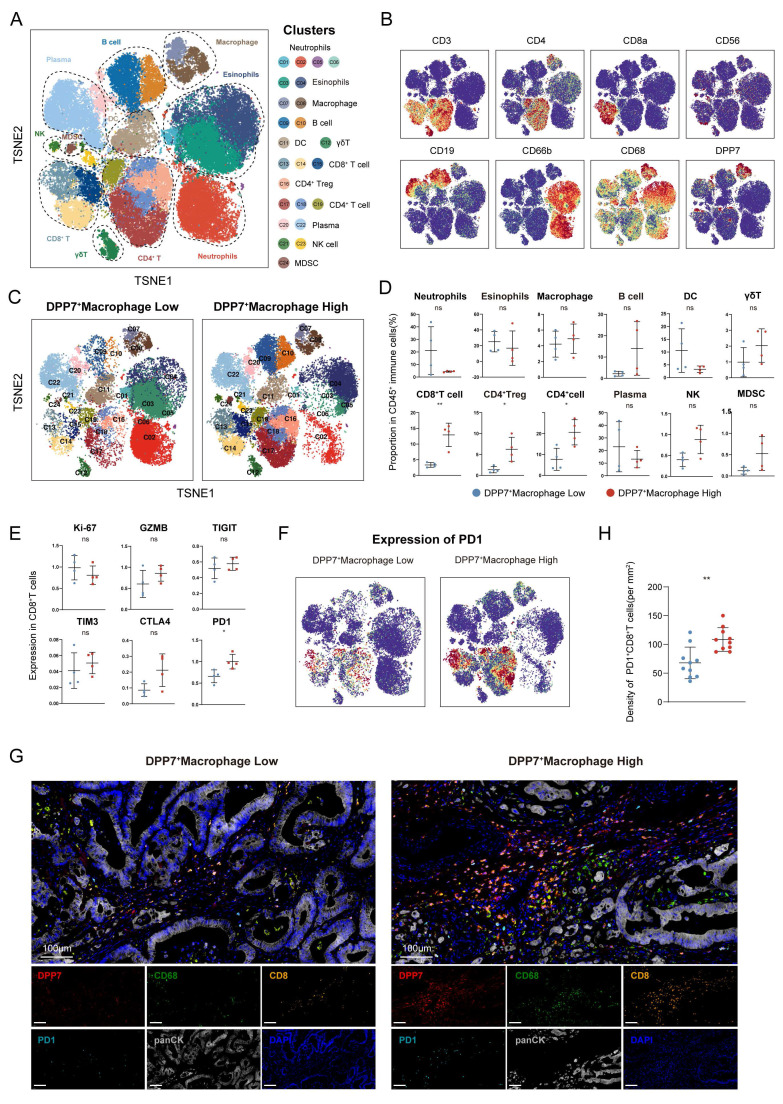
** DPP7^+^TAMs promote the formation of immunosuppressive TIME in CRC.** (A) t-SNE plot of CD45^+^ immune cells in the CRC analyzed by CyTOF across 8 patients. (B) t-SNE plot of CD45^+^ immune cells colored by the levels of CD3, CD4, CD8a, CD56, CD19, CD66b, CD68, and DPP7. (C) t-SNE plot of CD45^+^ immune cells divided by DPP7^+^macrophage low and high infiltration groups. (D) Proportion of immune cell types in CD45^+^ immune cells. (E) Expression of functional markers of CD8^+^T cells. (F) Expression of PD-1 in CD45^+^ immune cells. (G) Representative images of multiplex immunofluorescence in DPP7^+^TAMs-low (n = 10) and DPP7^+^TAMs-high (n = 10) human CRC tissues. (H) The correlations between DPP7^+^TAMs infiltration and PD-1^+^CD8^+^T cells. *p<0.05, **p<0.01, ***p<0.001, and ns = non-significant.

To explore the immunomodulatory role of DPP7^+^TAMs within the TIME, we conducted CyTOF analysis of 8 freshly resected CRC specimens. Unsupervised clustering of 24 distinct cellular subsets revealed predominant DPP7 expression in the TAMs (Fig.3A, B, Supplementary Fig.4A). Stratification of patients by DPP7 expression in TAMs revealed significant immunological remodeling in high DPP7^+^TAMs tumors (Fig.3C). Notably, these specimens exhibited significantly increased infiltration of both CD8^+^T cells and regulatory T cells (Tregs), accompanied by elevated accumulation of myeloid-derived suppressor cells (MDSCs) (Fig.3D). Functional profiling of CD8^+^T cells demonstrated that upregulation of the exhaustion marker PD-1 was positively associated with DPP7^+^TAMs infiltration (Fig.3E, F), which was further validated by mIF staining (Fig.3G, H) and cell-cell communication analysis (Supplementary Fig.4B).

**Figure 4:**
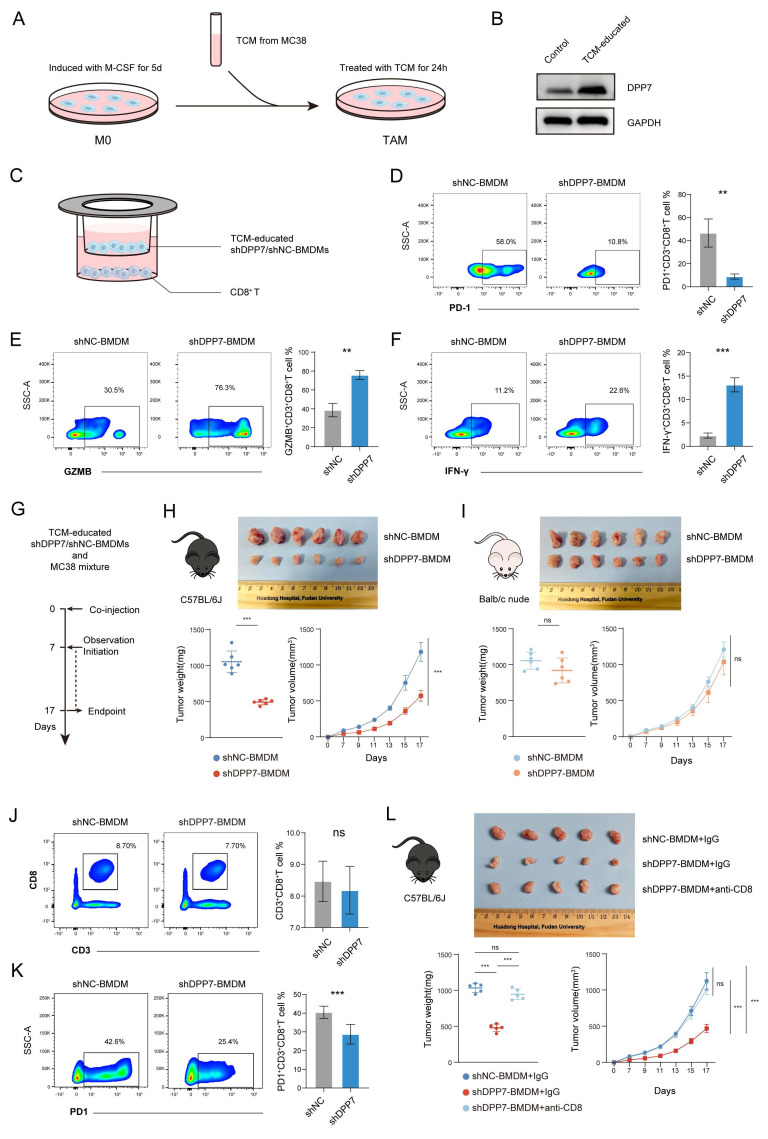
** DPP7^+^TAMs induce the exhaustion of CD8^+^T cells in CRC.** (A) Schematic representation of in vitro induction of TAM-like BMDMs. (B) WB results of DPP7 in untreated or TCM-educated BMDMs. (C) Schematic representation of co-culture of TCM-educated shDPP7/shNC-BMDMs and CD8^+^T cells. (D) Analysis of PD-1^+^CD8^+^T cells after co-culturing with BMDMs. (E) Analysis of GZMB^+^CD8^+^T cells after co-culturing with BMDMs. (F) Analysis of IFN-γ^+^CD8^+^T cells after co-culturing with BMDMs. (G) Schematic showing the schedule of shDPP7/shNC-transduced BMDMs and MC38 mixture co-injection to construct mice subcutaneous xenograft CRC tumor models. (H) Gross appearance, weight and volume of the subcutaneous CRC tumors in C57BL/6J mice (n=6 for each group). (I) Gross appearance, weight and volume of the subcutaneous CRC tumors in Balb/c nude mice (n=6 for each group). (J) Flow cytometry analyses of the tumor infiltration percentage of CD3^+^CD8^+^T cells in CD45^+^ cells from C57BL/6J mice. (K) Flow cytometry analyses of the tumor infiltration percentage of PD-1^+^ cells in CD3^+^CD8^+^T cells from C57BL/6J mice. (L) Gross appearance, weight and volume of the subcutaneous CRC tumors in C57BL/6J mice (n=5 for each group). *p<0.05, **p<0.01, ***p<0.001, and ns = non-significant.

In vitro and in vivo experiments were conducted to clarify the effect of DPP7^+^TAMs on CD8^+^T cells. BMDMs were isolated and exposed to tumor-conditioned medium (TCM) derived from murine CRC cells MC38 for 24h (Fig.4A). TAM-like BMDMs exhibited significantly increased DPP7 expression (Fig.4B). We established stable BMDM knockdown lines (shNC/shDPP7) after TCM induction (Supplementary Fig.4C) and conducted co-culture experiments with CD8^+^T cells (Fig.4C). DPP7 knockdown in BMDMs significantly reduced PD-1 expression (Fig.4D) and markedly upregulated the cytotoxic markers GZMB (Fig.4E) and IFN-γ (Fig.4F) in CD8^+^T cells. Furthermore, we established subcutaneous xenograft tumor models by co-implanting MC38 cells with TCM-educated shDPP7/shNC-transduced BMDMs (Fig.4G). DPP7-knockdown BMDMs demonstrated significant potent anti-tumor effects in immunocompetent C57BL/6J mice (Fig.4H), but not in T cell-deficient Balb/c nude mice (Fig.4I). Despite similar levels of CD8^+^T cell infiltration (Fig.4J), PD-1 expression was significantly downregulated of in the shDPP7-BMDM group (Fig.4K). In addition, tumor growth suppression mediated by DPP7 knockdown was abrogated following CD8^+^T cell depletion (Fig.4L). The efficiency of CD8^+^T cell depletion was detected using FCM (Supplementary Fig.4D). Taken together, DPP7^+^TAMs in the CRC TIME generally exhibit an immunosuppressive function, and may play a pivotal role in promoting tumor mainly by promoting immunosuppressive cells, especially exhausted CD8^+^T cells.

### 3.4 DPP7 induces immunosuppressive phenotype by promoting FAO in TAMs

**Figure 5:**
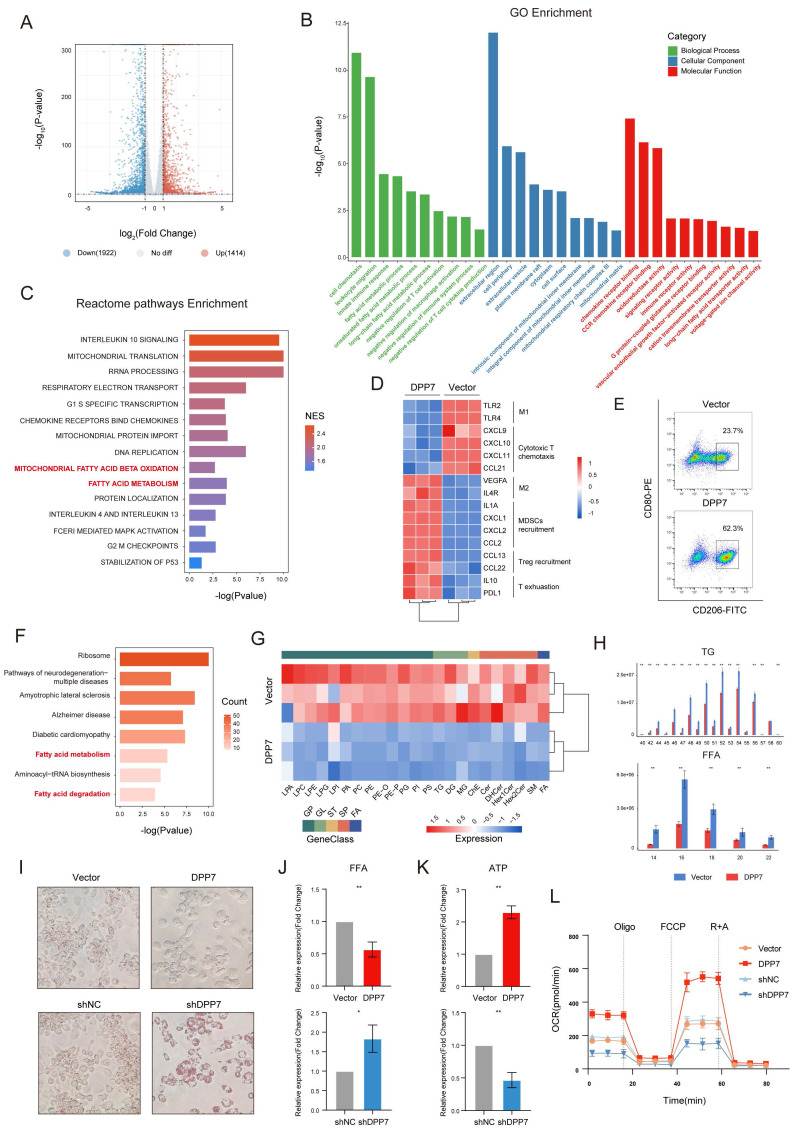
** DPP7 induces immunosuppressive phenotype by promoting FAO in TAMs.** (A) Volcano plot of differentially expressed genes (DEGs) analyzed by RNA-seq between Vector or DPP7 plasmid-transduced THP-1-differentiated macrophages. (B) GO enrichment analysis of DEGs between Vector and DPP7 groups. (C) Reactome pathways enrichment analysis of DEGs between Vector and DPP7 groups. (D) Heatmap of functional genes in Vector and DPP7 groups. (E) Flow cytometry analyses of CD80 and CD026 in Vector and DPP7 groups. (F) KEGG enrichment analysis of upregulated proteins interacting with DPP7 compared with IgG, based on LC-MS/MS results. (G) Heatmap of differentially abundant lipid metabolites between Vector and DPP7 groups. (H) Expression of triglycerides (TGs) and fatty acids (FFAs) with different carbon chains length between Vector and DPP7 groups. (I) Representative images showing the TGs content determined by oil red O staining. (J) Intracellular FFAs content detected by assay kit. (K) Intracellular ATP content detected by assay kit. (L) The OCR was measured at baseline and in response to incubation with oligomycin (Oligo), FCCP (Carbonyl Cyanide 4-(trifluoromethoxy) phenylhydrazone), and rotenone plus antimycin A (R + A) in THP-1-differentiated macrophages. *p<0.05, **p<0.01, ***p<0.001, and ns = non-significant.

To elucidate the mechanism of the DPP7-driven immunosuppressive phenotype, we conducted RNA sequencing of DPP7-overexpressing THP-1-derived macrophages versus the control. RNA sequencing revealed 1,414 upregulated (top 5 genes: SERPINA11, SERPINA9, SMACRO, C1QL2, TMEM185AP1) and 1,922 downregulated (top 5 genes: MYH6, GRID1, OR52I2, KLRC1, PRR33) genes (Fig.5A), with GO enrichment analysis implicating fatty acid metabolism, mitochondrial dynamics, immune modulation, and chemokine signaling as key altered pathways (Fig. 5B). Reactome pathway analysis further demonstrated DPP7-mediated regulation of inflammatory interleukins and fatty acid metabolic reprogramming, particularly in FAO (Fig.5C). To better understand the mechanism by which DPP7^+^TAMs contribute to the formation of an immunosuppressive TIME, we further focused on the changes in specific cytokine expression profiles. Notably, DPP7 overexpression induced a characteristic immunosuppressive signature: suppression of M1 markers (TLR2/4) and cytotoxic T cell-recruiting chemokines (CXCL9/10/11, CCL21), coupled with upregulation of M2 markers (VEGFA, IL4R), MDSCs, and Treg recruitment factors (IL1A, CXCL1/2, CCL2/13/22), and T cell exhaustion mediators (IL10, PD-L1) (Fig.5D), which were all validated by qPCR (Supplementary Fig.5A). Flow cytometry results confirmed that the enhanced M2 polarization phenotype was caused by DPP7 overexpression (Fig.5E, Supplementary Fig.5B, C). Besides, prototypical M2-polarization markers (CD163, CD204, and CD206) and immunosuppressive features (PD-L1 upregulation with concomitant M1 marker CD86 suppression) were also observed in DPP7^+^TAMs compared with DPP7^-^TAMs based on CyTOF results (Supplementary Fig.5D, E). In addition, we observed higher levels of M2-polarized genes in DPP7^+^TAMs, which supported that DPP7^+^TAMs exhibited an M2 polarization phenotype (Supplementary Fig.6). Furthermore, considering that DPP7 has been reported to promote the apoptosis of lymphocytes^18^, ^19^, we therefore performed apoptosis assays on macrophages, and the results showed that DPP7 overexpression did not affect the apoptosis process (Supplementary Fig.5F). These experimental results suggest that DPP7 promotes the transformation of macrophages into the M2 phenotype.

To explore how DPP7 promotes M2 polarization of macrophages, we performed LC-MS/MS to analyze DPP7-interacting proteins obtained by Co-IP experiments. By interlacing the RNA-seq results with the LC-MS/MS analysis, we found that the overexpression of DPP7 may significantly affect fatty acid metabolism in macrophages (Fig.5F). It has been reported that FAO-driven metabolic flux activates STAT6 via mitochondrial oxidative phosphorylation, SHP1 dephosphorylation, ROS generation, and JAK1 phosphorylation. This cascade reinforces M2 polarization by inducing markers such as arginase 1 (Arg1) and CD206^29^-^31^. Therefore, we hypothesized that DPP7 orchestrated immunosuppression by participating in FAO regulation. To validate whether DPP7 affected FAO, lipid-targeted metabolomic sequencing was conducted and revealed profound depletion of triglycerides (TGs) and fatty acids (FAs) with different carbon chains in DPP7-overexpressing cells (Fig.5G, H). Decreased intracellular TG and FFA levels (Fig.5I, J) and increased ATP generation (Fig.5K) was observed in THP-1-differentiated macrophages. Similar changes were observed in BMDMs (Supplementary Fig.5G-I). Functional metabolic assays demonstrated DPP7-dependent augmentation of oxygen consumption rates (OCR) (Fig.5L). In all, these results mechanistically link DPP7 activity to enhanced mitochondrial FAO capacity, thus promoting an immunosuppressive phenotype.

### 3.5 DPP7 enhances FAO by interacting with CPT1A and reducing its ubiquitination-dependent degradation

**Figure 6:**
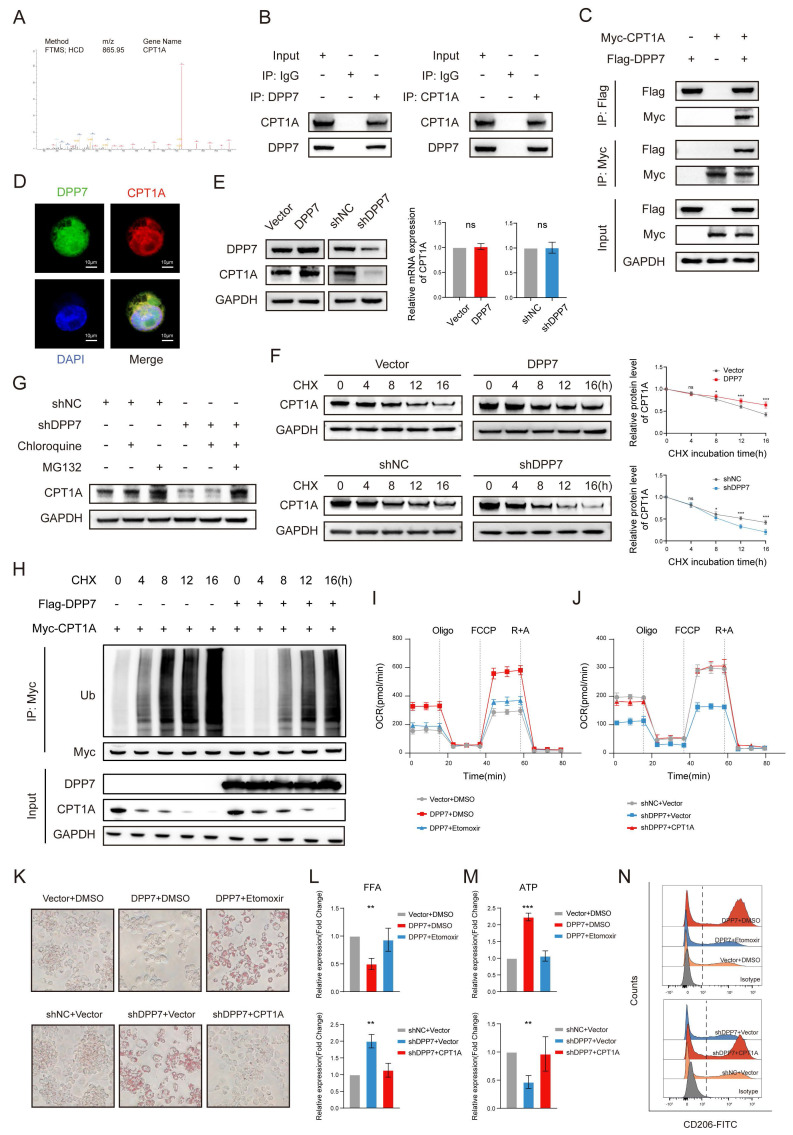
** DPP7 enhances FAO by interacting with CPT1A and reducing its ubiquitination-dependent degradation.** (A) The peptide spectrum of CPT1A, as determined by LC‒MS/MS analysis of DPP7 immunoprecipitates. (B) Co-IP assays of DPP7 and CPT1A performed in THP-1-differentiated macrophages. (C) Co-IP assays of DPP7 and CPT1A performed in HEK293T cells with overexpression of DPP7 or CPT1A. (D) The colocalization of DPP7 (green) and CPT1A (red) detected by IF staining. (E) The expression of CPT1A in DPP7 overexpression or knockdown macrophages. (F) Evaluation of the degradation of CPT1A in DPP7 overexpression or knockdown macrophages with incubation of cycloheximide (CHX). (G) The expression of CPT1A in macrophages with treated with MG132 or chloroquine. (H) The levels of time-course ubiquitination of CPT1A in HEK293T cells. (I) The OCR was measured in macrophages treated with or without DPP7 overexpression and Etomoxir. (J) The OCR was measured in macrophages treated with or without DPP7 knockdown and CPT1A overexpression. (K) Representative images showing the TGs content determined by oil red O staining. (L) Intracellular FFAs content detected by assay kit. (M) Intracellular ATP content detected by assay kit. (N) Flow cytometry analyses of CD206 in macrophages. *p<0.05, **p<0.01, ***p<0.001, and ns = non-significant.

To reveal the concrete mechanism, we reviewed DDP7-interacting proteins involved in fatty acid metabolism and fatty acid degradation pathways, and focused on CPT1A as it ranked first in the upregulated protein list compared with IgG (Fig.6A). Other upregulated proteins were listed in Supplementary Table 4. CPT1A is a mitochondrial enzyme that regulates FAO^32^. Notably, CPT1A is significantly overexpressed in metastatic CRC^33^, ^34^ and mechanistically linked to M2 polarization through FAO activation in TAMs^29^-^31^, ^35^. Experimental results confirmed robust DPP7-CPT1A interaction through Co-IP in both endogenous expression (Fig.6B) and exogenous overexpression systems (Fig.6C), with cellular colocalization demonstrated by confocal microscopy (Fig.6D). Intriguingly, DPP7 overexpression or knockdown modulated CPT1A protein levels without affecting mRNA expression (Fig.6E, Supplementary Fig.7A), suggesting post-translational regulation. Cycloheximide (CHX) chase assays revealed that DPP7 overexpression reduced CPT1A degradation, whereas DPP7 knockdown accelerated (Fig.6F). Pharmacological inhibition using chloroquine (a lysosomal acidification inhibitor) and MG132 (a proteasomal activity blocker) demonstrated that CPT1A degradation predominantly occurred via the ubiquitin-proteasome system (Fig.6G). Crucially, DPP7 overexpression substantially attenuated CPT1A time-course ubiquitination (Fig.6H), establishing its role in the modulation of ubiquitin-mediated proteolysis. Next, etomoxir was added to DPP7-overexpressing macrophages to inhibit CPT1A and rescued the FAO hyperactivation phenotype (Fig.6I), TG/FFA levels (Fig.6K, L), ATP overproduction (Fig.6M), and M2 polarization markers (Fig.6N, Supplementary Fig.7B). Reciprocally, CPT1A overexpression in DPP7-knockdown cells restored FAO capacity (Fig.6J), metabolic substrate utilization (Fig.6K, L), ATP production (Fig.6M), and M2 polarization (Fig.6N, Supplementary Fig.7C). These results demonstrate that DPP7 promotes FAO of TAMs by reducing the ubiquitination-induced degradation of CPT1A, which leads TAMs to the M2 polarization state.

### 3.6 DPP7 reduces the ubiquitination-induced degradation of CPT1A by interacting with CPT1A in a mutually exclusive manner with TRIM25

**Figure 7:**
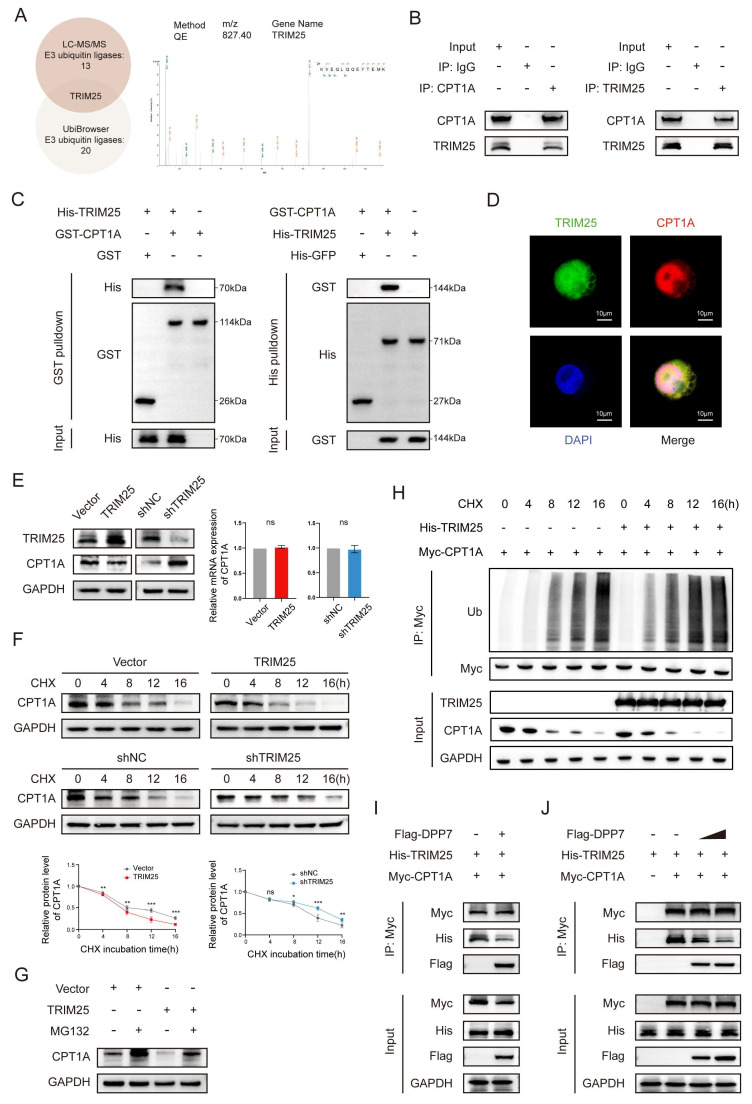
** DPP7 reduces the ubiquitination-induced degradation of CPT1A by interacting with CPT1A in a mutually exclusive manner with TRIM25.** (A) The peptide spectrum of TRIM25, as the intersection of LC‒MS/MS analysis of CPT1A immunoprecipitate and prediction result from UbiBrowser website. (B) Co-IP assays of TRIM25 and CPT1A performed in THP-1-differentiated macrophages. (C) Pulldown assays of TRIM25 and CPT1A. (D) The colocalization of TRIM25 (green) and CPT1A (red) detected by IF staining. (E) The expression of CPT1A in TRIM25 overexpression or knockdown THP-1-differentiated macrophages. (F) Evaluation of the degradation of CPT1A in TRIM25 overexpression or knockdown macrophages with the application of cycloheximide (CHX). (G) The expression of CPT1A in macrophages with treated with MG132 or chloroquine. (H) The levels of time-course ubiquitination of CPT1A in HEK293T cells. (I) Co-IP analysis of the binding of TRIM25 to CPT1A in HEK293T cells transfected with or without Flag-DPP7. (J) Co-IP analysis of the binding of TRIM25 to CPT1A in HEK293T cells treated with different concentrations of exogenous Flag-DPP7. *p<0.05, **p<0.01, ***p<0.001, and ns = non-significant.

To delineate the ubiquitination-induced degradation of CPT1A, we performed LC-MS/MS to identify the CPT1A-coupled proteins. Cross-referencing these candidate proteins with UbiBrowser-predicted E3 ubiquitin ligases (Supplementary Fig.8A), we focused on TRIM25 (tripartite motif-containing 25) (Fig. 7A), which plays a pivotal role in various cancers by inducing ubiquitination^36^-^38^. We confirmed the TRIM25-CPT1A interaction through reciprocal Co-IP (Fig.7B), pull-down assays (Fig.7C), and cellular colocalization analysis (Fig.7D). TRIM25 overexpression or knockdown modulated CPT1A protein level without transcriptional alterations (Fig.7E and Supplementary Fig.8B). CHX chase assays demonstrated that TRIM25 overexpression accelerated CPT1A degradation (Fig.7F), whereas proteasomal inhibition via MG132 effectively blocked this process (Fig.7G). Critically, overexpression of TRIM25 significantly increased CPT1A time-course ubiquitination (Fig.7H). These results indicate that TRIM25 is an E3 ubiquitination ligase that mediates the ubiquitination degradation of CPT1A in TAMs.

Reviewing the LC-MS/MS results of DPP7-binding proteins, we did not detect any interaction between DPP7 and TRIM25. Based on these previous findings, we hypothesized that DPP7 inhibited TRIM25 by binding to CPT1A. Intriguingly, increasing DPP7-CPT1A binding diminished the TRIM25-CPT1A interaction (Fig.7I). Sequential immunoprecipitation experiments quantitatively established an inverse correlation between the DPP7-CPT1A interaction and TRIM25-CPT1A complex formation (Fig.7J). At the same time, we did not find a significant decrease in the protein expression level of TRIM25 with an increase in DPP7 expression, which indicated that DPP7 did not directly affect TRIM25 (Fig.7I, J). Therefore, as a competitive binder, DPP7 sterically hinders TRIM25-mediated ubiquitination, resulting in high expression of CPT1A.

### 3.7 DPP7 knockdown in BMDMs enhances the efficacy of PD-1 blockade and reverse the immunosuppressive TIME

**Figure 8:**
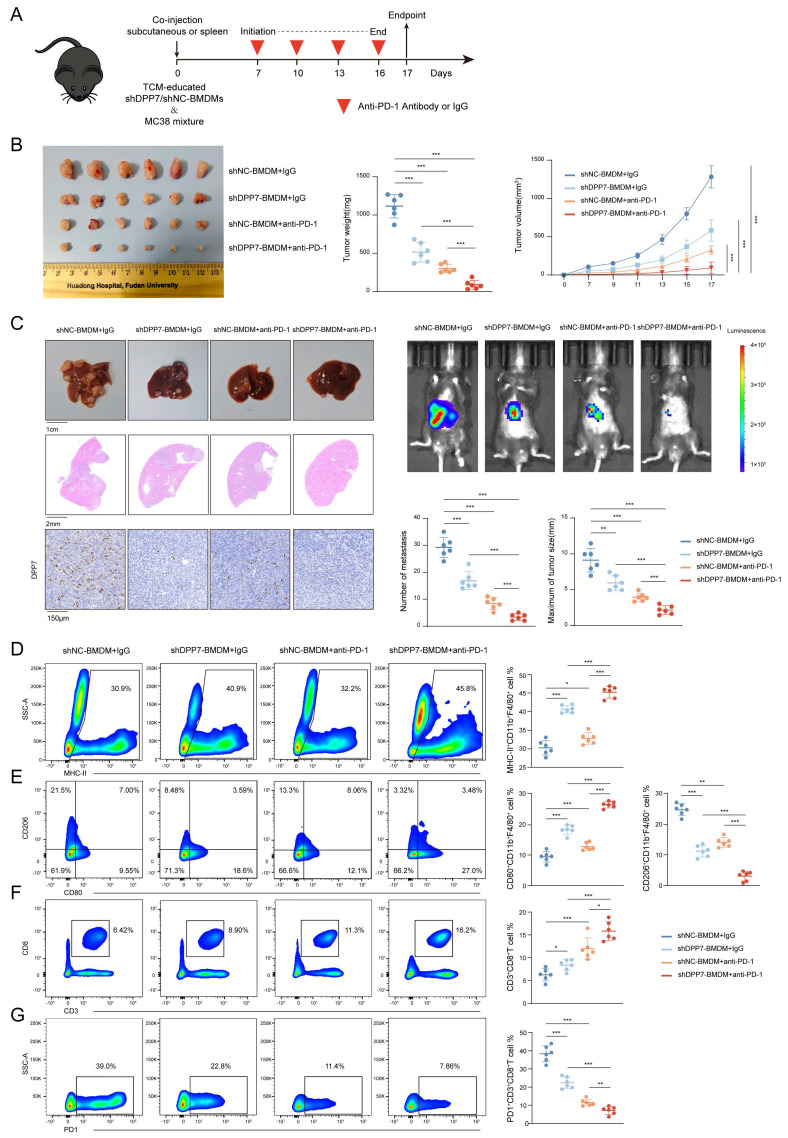
** DPP7 knockdown in BMDMs enhances the efficacy of PD-1 blockade and reverse the immunosuppressive TIME.** (A) Schematic showing the schedule of TCM-educated shDPP7/shNC-BMDMs and MC38 mixture co-injection to construct mice subcutaneous xenograft and liver metastasis tumor models on the day 0, followed by anti-PD-1 or IgG treatment starting on the day 7, once every 3 days for a total of 4 doses. (B) Gross appearance, weight and volume of the subcutaneous CRC tumor models in different treatment groups (n=6 for each group). (C) Gross appearance, HE staining, IHC, in vivo imaging, number of metastasis and maximum of tumor size of liver metastasis models in different treatment groups (n=6 for each group). (D) Flow cytometry analyses of the tumor infiltration percentage of MHC-II^+^ cells in CD11b^+^F4/80^+^ macrophages. (E) Flow cytometry analyses of the tumor infiltration percentage of CD80^+^ and CD206^+^ cells in CD11b^+^F4/80^+^ macrophages. (F) Flow cytometry analyses of the tumor infiltration percentage of CD3^+^CD8^+^T cells in CD45^+^ cells. (G) Flow cytometry analyses of the tumor infiltration percentage of PD-1^+^ cells in CD3^+^CD8^+^T cells. *p<0.05, **p<0.01, ***p<0.001, and ns = non-significant.

Based on these findings, we evaluated the therapeutic potential of DPP7 inhibition in augmenting immunotherapy for CRC. We established subcutaneous xenograft and liver metastasis mouse tumor models by co-injecting MC38 cells with TCM-educated shDPP7/shNC-transduced BMDMs, followed by anti-PD-1 or IgG treatment (Fig. 8A). Therapeutic intervention with shDPP7-BMDMs, anti-PD-1 monotherapy, and the combination demonstrated significant suppression of both subcutaneous tumor growth and liver metastatic burden compared with the control (Fig.8B, C). Strikingly, the combination regimen exhibited the best anti-tumor efficacy (Fig.8B, C), indicating the synergistic enhancement of PD-1 blockade through DPP7 suppression in BMDMs. Furthermore, flow cytometry was used to investigate the TIME. Combination-treated tumors displayed TAMs with elevated MHC-II and CD80 expression but decreased CD206 expression (Fig.8D, E), indicative of enhanced antigen-presenting capacity and diminished immunosuppressive polarization. Therapeutic synergy increased tumor-infiltrating CD8^+^T lymphocytes and reduced exhausted PD-1^+^CD8^+^T cell subsets (Fig.8F, G). Furthermore, we observed that shDPP7-BMDMs led to an upregulation of PD-L1 expression within the tumors (Supplementary Fig.9A). Notably, the combination therapy group exhibited a further elevation in PD-L1 levels (Supplementary Fig.9A, B), suggesting a potential mechanism underlying the synergistic effect observed with PD-1 blockade combined with DPP7 suppression. Overall, we consider that DPP7 knockdown in BMDMs may potentiate PD-1 blockade efficacy through reprogramming of the TIME.

## 4. Discussion

Although PD-1 blockade has achieved limited success in solid tumors, accumulating clinical evidence implicates immunosuppressive networks within the TIME as the principal bottleneck constraining immunotherapy efficacy. Macrophages, the major components of immune cells in the TIME, have emerged as pivotal therapeutic targets for synergizing with ICIs. Current TAM-targeted strategies coalesce into three paradigms: (1) depletion of existing TAMs, (2) inhibition of monocyte/TAM recruitment, and (3) reprogramming them toward an M1-like anti-tumor phenotype^39^-^42^. However, indiscriminate TAMs elimination risks ablate their dual roles as phagocytes and antigen-presenting cells (APCs), thereby compromising innate and adaptive anti-tumor immunity^13^. Compensatory infiltration of tumor-associated neutrophils (TANs) frequently mediates therapeutic resistance following TAMs depletion, while therapy-terminated rebound mobilization of bone marrow-derived monocytes may paradoxically fuel metastatic dissemination^43^, ^44^. Crucially, the inherent plasticity of TAMs, despite their pro-tumorigenic default state, retains latent potential for functional re-education into tumoricidal M1 effectors capable of orchestrating cytotoxic T cell responses and suppressing oncogenesis^28^, ^45^. This functional duality underscores macrophage reprogramming as the most viable strategy, and the identification of novel TAM targets represents a critical frontier in immunotherapy.

Emerging evidence suggests that elevated DPP7 expression correlates with unfavorable clinical outcomes in various malignancies, however, current research remains limited. Existing studies primarily concentrate on clinicopathological correlations^20^, ^22^ or direct tumorigenic effects of DPP7^21^, ^23^, while critical gaps persist in understanding its cellular expression pattern and function in the CRC TIME. Our comprehensive investigation combining scRNA sequencing, IHC, IF, and CyTOF, revealed a macrophage marker DPP7, with low expression levels detected in neoplastic cells and other mesenchymal and immune subsets. Notably, DPP7^+^TAMs demonstrated significantly enhanced infiltration in malignant tissues than in normal tissues, particularly in metastatic lesions. Clinical correlation analyses further established that high DPP7^+^TAM infiltration served as a prognostic indicator of worse OS. This study is the first to report a distinct DPP7^+^TAM subpopulation, and delineate its clinical significance as a novel poor prognostic biomarker in CRC.

Metabolic reprogramming is a hallmark of cancer biology^46^. To coexist with rapidly proliferating tumor cells, TAMs undergo extensive lipid metabolic reprogramming, which is marked by amplified fatty acid uptake and preferential utilization of FAO over glycolysis as their ATP resources^29^. The key to this process is CPT1A, a rate-limiting mitochondrial enzyme governing FAO^32^. Elevated CPT1A expression, clinically associated with metastatic progression in CRC^33^, ^34^, underscores its functional importance in disease aggressiveness. Additionally, TAMs exhibit pronounced CPT1A upregulation^29^, ^30^. Mechanistically, the FAO-driven metabolic flux activates STAT6 via mitochondrial oxidative phosphorylation, SHP1 dephosphorylation, ROS generation, and JAK1 phosphorylation. This cascade reinforces M2 polarization by inducing markers such as Arg1 and CD206, which can be reversed by FAO inhibition^35^. While targeting CPT1A to disrupt FAO and reprogram TAMs holds therapeutic promise, the clinical application of the canonical CPT1A inhibitor etomoxir remains constrained by off-target organ toxicity and metabolic disturbances^47^-^49^.

Our findings revealed a novel regulatory axis: DPP7 in TAMs physically interacts with CPT1A to competitively inhibit TRIM25-mediated ubiquitination degradation of CPT1A, thereby stabilizing FAO activity and driving M2 polarization. This discovery positioned DPP7 as a precise strategy target to subvert lipid metabolic reprogramming in TAMs, offering a potential alternative to direct CPT1A inhibition. Unfortunately, there are currently no commercially available DPP7 inhibitors. While some reported inhibitors, such as Talabosta and Talabostat mesylate, can inhibit DPP7 at certain concentrations, they are not primarily used as DPP7 inhibitors due to their lack of selectivity. Furthermore, as our study demonstrates, DPP7 is primarily but not exclusively expressed on macrophages. Considering that the inhibition of DPP7 induces apoptosis in resting lymphocytes^19^, the expression pattern also poses a challenge for cell-specific targeting. Therefore, achieving both structural specificity and cellular specificity remains a key hurdle in developing effective DPP7 inhibitors. Meanwhile, based on our mechanistic findings, the DPP7-mediated reduction in CPT1A ubiquitination may not necessarily depend on DPP7 enzymatic activity. Alternative DPP7- targeting strategies could include disrupting the DPP7-CPT1A protein- protein interaction (PPI inhibitors) or employing targeted protein degradation (TPD), which may offer higher selectivity.

The immune cell infiltration landscape fundamentally shapes therapeutic responses to immunotherapy^31^. Contemporary classifications stratify TIME into three distinct states: immune-excluded, immune-inflamed, and tertiary lymphoid structure-enriched^9^. Our mechanistic investigation identified that DPP7^+^TAMs drove the formation of an immunosuppressive niche by recruiting and promoting exhausted immune cells. Thus, we characterized DPP7^+^TAMs with high TIME as immune-inflamed, paradoxically enriched with both terminally exhausted lymphocytes and immunosuppressive leukocytes, which are conventionally associated with improved immunotherapy responses^9^. The combination therapy significantly suppressed both subcutaneous tumor growth and liver metastatic burden in our experiments, thus we considered that the combination therapy activated the local and systemic immunity successfully, which validated our hypothesis as well. Interestingly, we found that the administration of shDPP7-BMDMs significantly activated local tumor immunity, while concurrently stimulated the expression of PD-L1 by the tumor cells, which may represent a compensatory immun resistance mechanism following the initial immune activation^11^. The concomitant use of anti-PD-1 therapy effectively blocked this secondary inhibitory signal, potentially explaining the synergistic effect observed between shDPP7-BMDMs and anti-PD-1 blockade. This finding provides a potential approach for improving the efficacy of immunotherapy in CRC patients.

Therapeutic targeting of DPP7 remains an uncharted frontier in clinical oncology, with no commercially available inhibitors currently undergoing clinical evaluation. Two critical challenges emerge:(1) developing agents with sufficient structural and cellular specificity, and pharmacokinetic stability to disrupt DPP7-mediated immunosuppression, and (2) establishing optimal combinatorial strategies with existing immune checkpoint inhibitors through rational therapeutic sequencing. Furthermore, the observed synergy between DPP7 modulation and PD-1 blockade in murine models requires further validation in larger-scale prospective clinical cohorts.

## 5. Conclusion

**Figure 9:**
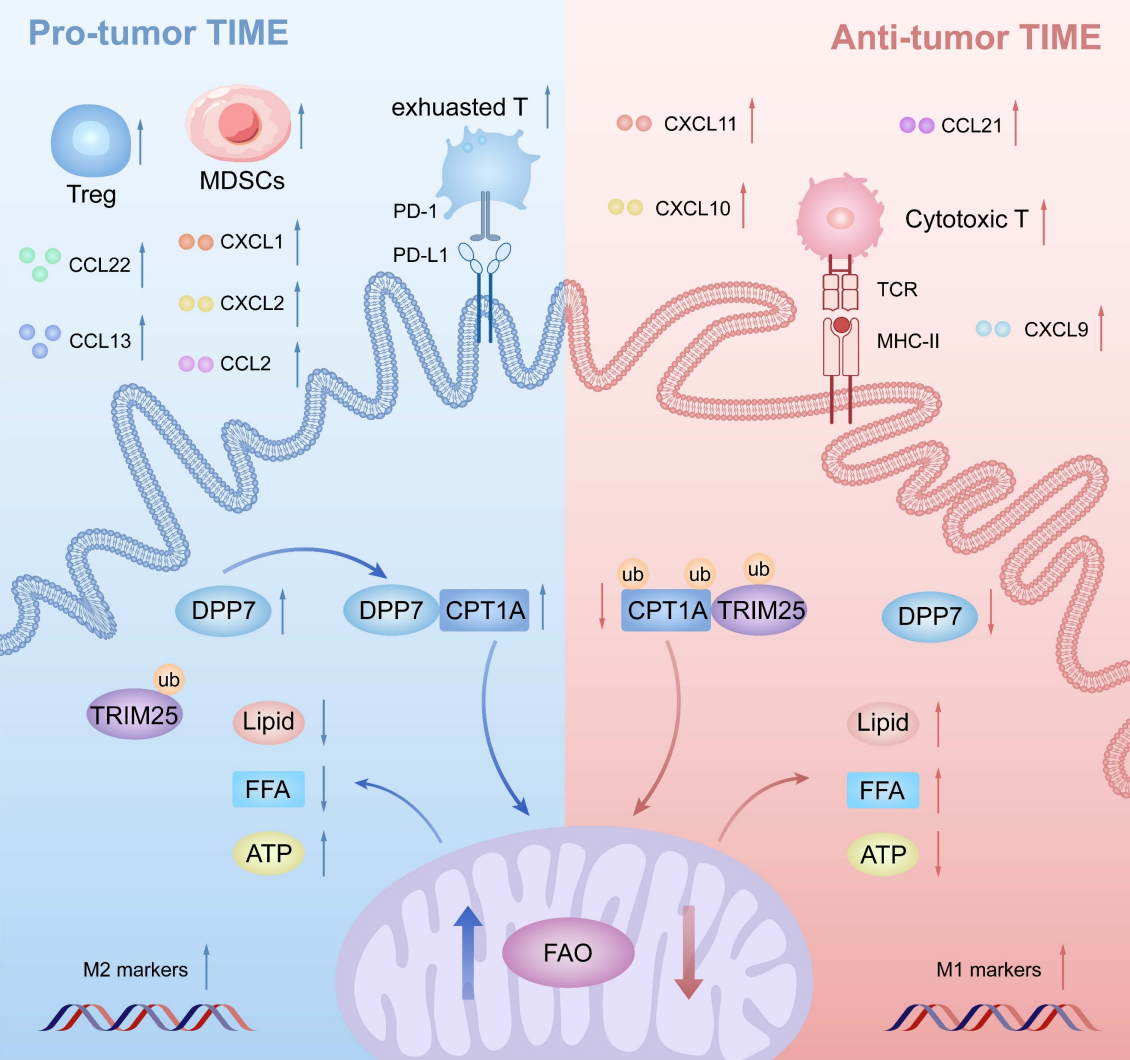
** Schematic illustration of DPP7-mediated TAMs metabolic reprogramming in CRC.** Mechanistically in TAMs, DPP7 competitively binds to CPT1A, antagonizing TRIM25-mediated ubiquitination and subsequent proteasomal degradation, thereby enhancing FAO-dependent metabolic reprogramming that drives M2 polarization. This metabolic shift concurrently impairs TAMs phagocytic capacity while promoting recruitment of immunosuppressive cell populations including exhausted CD8^+^T cells, Tregs, and MDSCs, ultimately establishing an immune-evasive TIME.

DPP7 is mainly expressed in macrophages, and DPP7^+^TAMs are strongly associated with an adverse prognosis in CRC. Mechanistically, DPP7 enhances FAO to promote the M2-polarized phenotype in TAMs, thus leading to an immunosuppressive TIME (Fig.9). Knockdown DPP7 in TAMs restores the phagocytic ability of macrophages, thereby remodeling the immunosuppressive tumor microenvironment. Targeting macrophage DPP7 could synergize with immunotherapy, suggesting that combination therapy may represent a novel therapeutic strategy for CRC patients.

## Supplementary Material

Supplementary figures and tables.

## Figures and Tables

**Table 1:** Cox regression analysis for OS of Huadong cohort

	Univariate		Multivariate	
	HR (95% CI)	P	HR (95% CI)	P
Gender		0.243		
Female	1 (reference)			
Male	0.78(0.52-1.18)			
Age				
≤60	1 (reference)	0.451		
>60	1.29(0.67-2.49)			
Preoperative serum CEA (ng/ml)		0.965		
≤5	1 (reference)			
>5	0.99(0.66-1.50)			
Tumor location				
Left-sided colon	1 (reference)		1 (reference)	
Right-sided colon	1.80(1.08-2.98)	0.024	1.73(1.03-2.89)	0.037
Rectum	0.88(0.53-1.45)	0.611	0.94(0.57-1.58)	0.827
Tumor size (cm)		0.095		
≤4	1 (reference)		1 (reference)	0.632
>4	1.42(0.94-2.14)		1.11(0.72-1.70)	
Histology		0.836		
Non-adenocarcinoma	1 (reference)			
Adenocarcinoma	0.93(0.47-1.85)			
Differentiation		0.831		
Well/moderately	1 (reference)			
Poorly/undifferentiated	0.93(0.50-1.76)			
T stage		0.144		
T1-2	1 (reference)			
T3-4	1.40(0.89-2.21)			
N stage		0.005		0.026
N0	1 (reference)		1 (reference)	
N1-2	1.82(1.20-2.76)		1.63(1.06-2.52)	
M stage		<0.001		<0.001
M0	1 (reference)		1 (reference)	
M1	3.06(2.00-4.67)		2.38(1.53-3.71)	
MMR status		0.955		
pMMR	1 (reference)			
dMMR	0.98(0.43-2.24)			
Density of DPP7^+^TAMs		0.012		0.037
Low	1 (reference)		1 (reference)	
High	1.72(1.13-2.61)		1.58(1.03-2.43)	

CEA, carcinoembryonic antigen; MMR, mismatch repair.
