# Supplementary material for: DPP7 promotes fatty acid β-oxidation in tumor-associated macrophages and determines immunosuppressive microenvironment in colorectal cancer
Source: Int J Biol Sci. 2025 Oct 1;21(14):6305–25. doi: 10.7150/ijbs.117909 (PMC12594597; doi:10.7150/ijbs.117909)
Supplement: Supplementary file 1 — Supplementary figures and tables. [file ijbsv21p6305s1.pdf]

Supplementary Materials

The Supplementary Data consist of:

Supplementary Figure 1-9

Supplementary Table 1-9

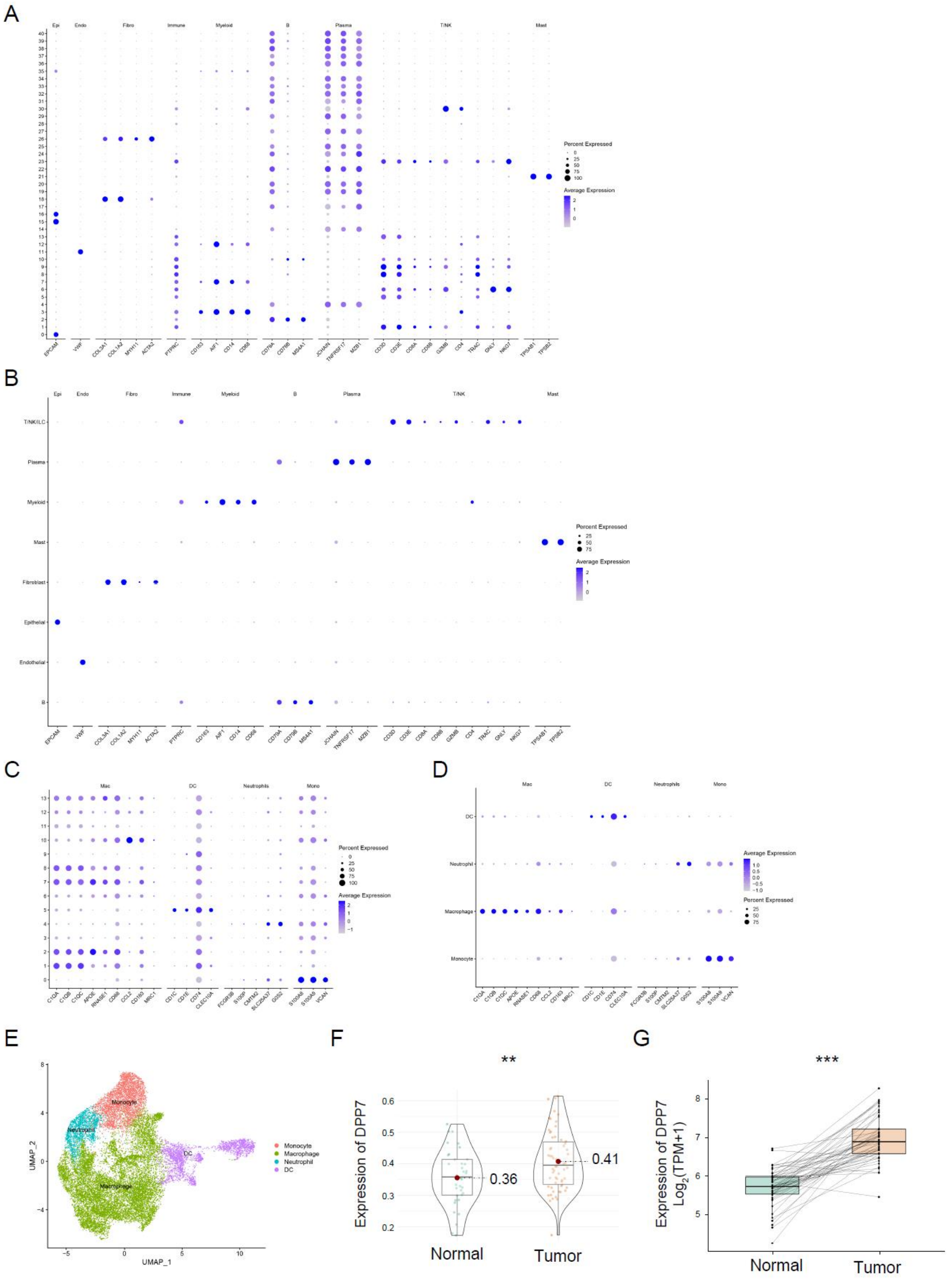

Supplementary Figure 1. Single-cell RNA sequencing annotation (GSE178341). (A) Expression of canonical cellular markers across all subclusters. (B) Marker expression across following annotated cell-type. (C) Myeloid cell markers across myeloid

subclusters. (D) Myeloid markers distribution within annotated myeloid subsets. (E) t-SNE visualization of annotated myeloid cell populations. (F) The expression of DPP7 in normal tissues and CRC tissues based on scRNA-seq dataset. (G) The expression of DPP7 in paired normal tissues and CRC tissues based on TCGA cohort. *p<0.05, **p<0.01, ***p<0.001, and ns = non-significant.

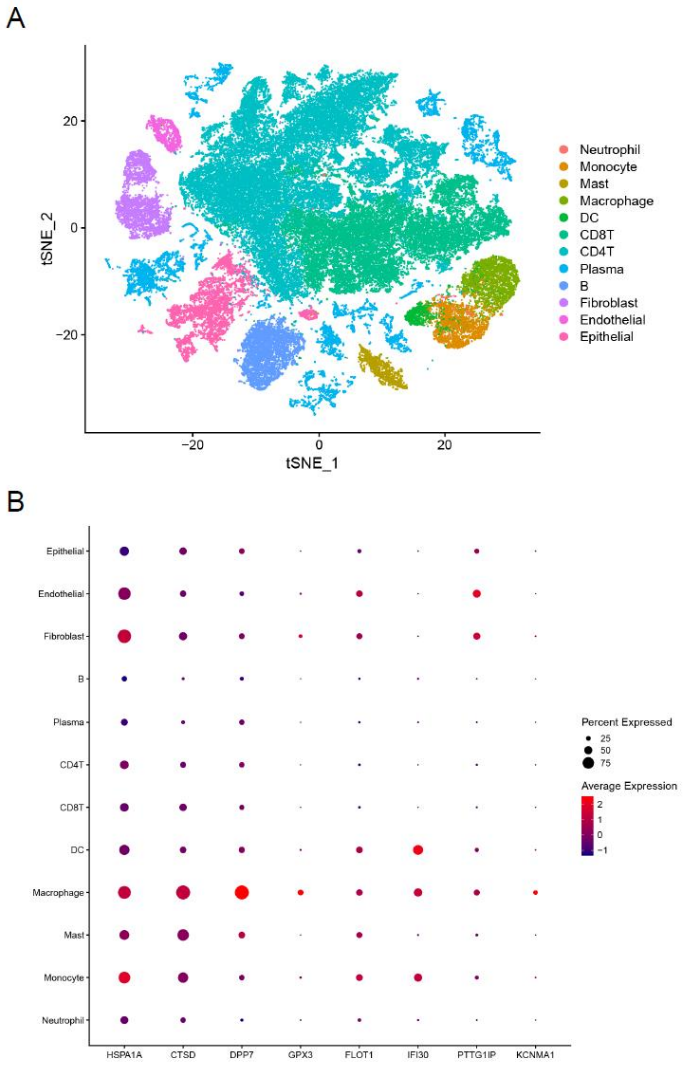

Supplementary Figure 2. Single-cell RNA sequencing validation (PRJNA748525).

(A) t-SNE visualization of all annotated cell types. (B) Dot plot of eight valuable macrophage genes across all cell types.

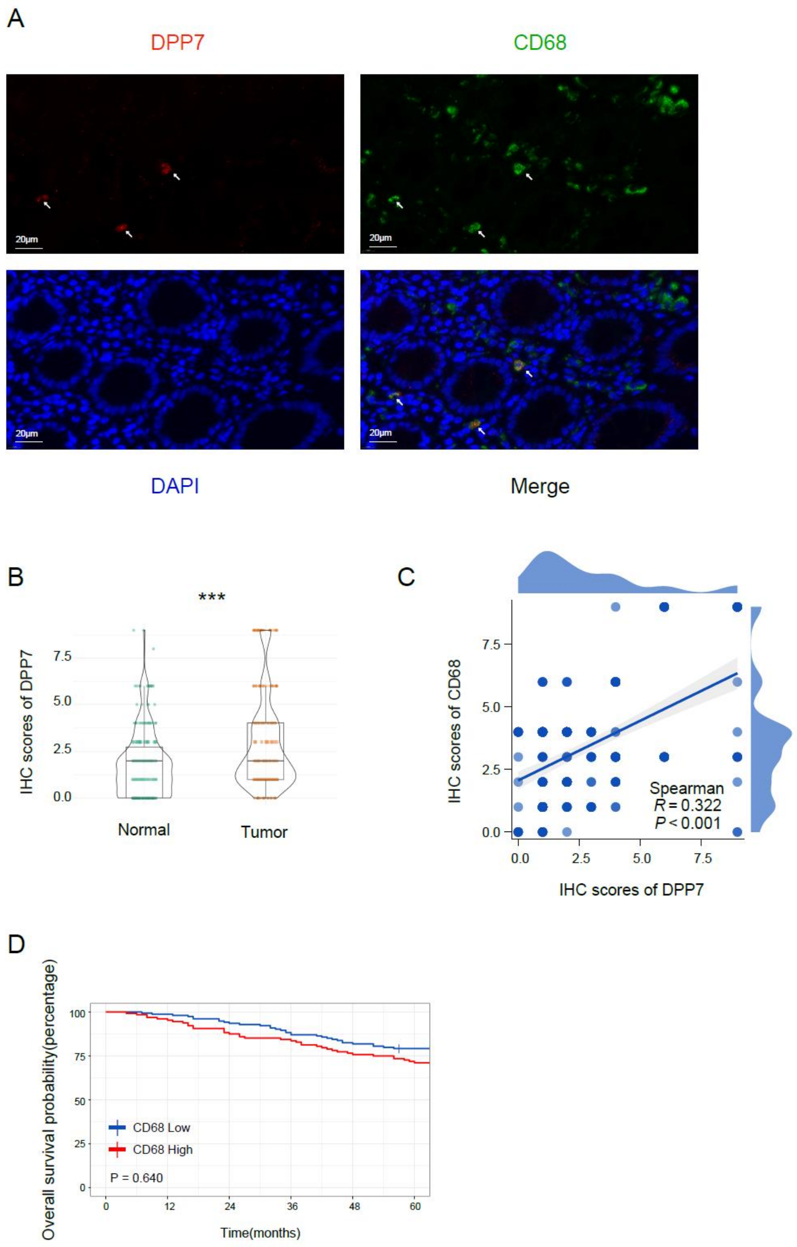

Supplementary Figure 3. Infiltration of DPP7+TAMs is significantly correlated with prognosis in patients with CRC. (A) Representative images of IF co-staining of DPP7 (red) and CD68 (green) as DPP7^{+}TAMs in normal tissues from the Huadong hospital cohort. (B) The IHC scores expression of DPP7 in normal tissues and CRC tissues from the Huadong patient cohort. (C) Spearman correlation analysis of IHC scores of CD68 and DPP7. (D) OS curves for CRC patients with high and low expression of CD68 based on IHC staining in the Huadong hospital cohort (n=282). *p<0.05, **p<0.01, ***p<0.001, and ns = non-significant.

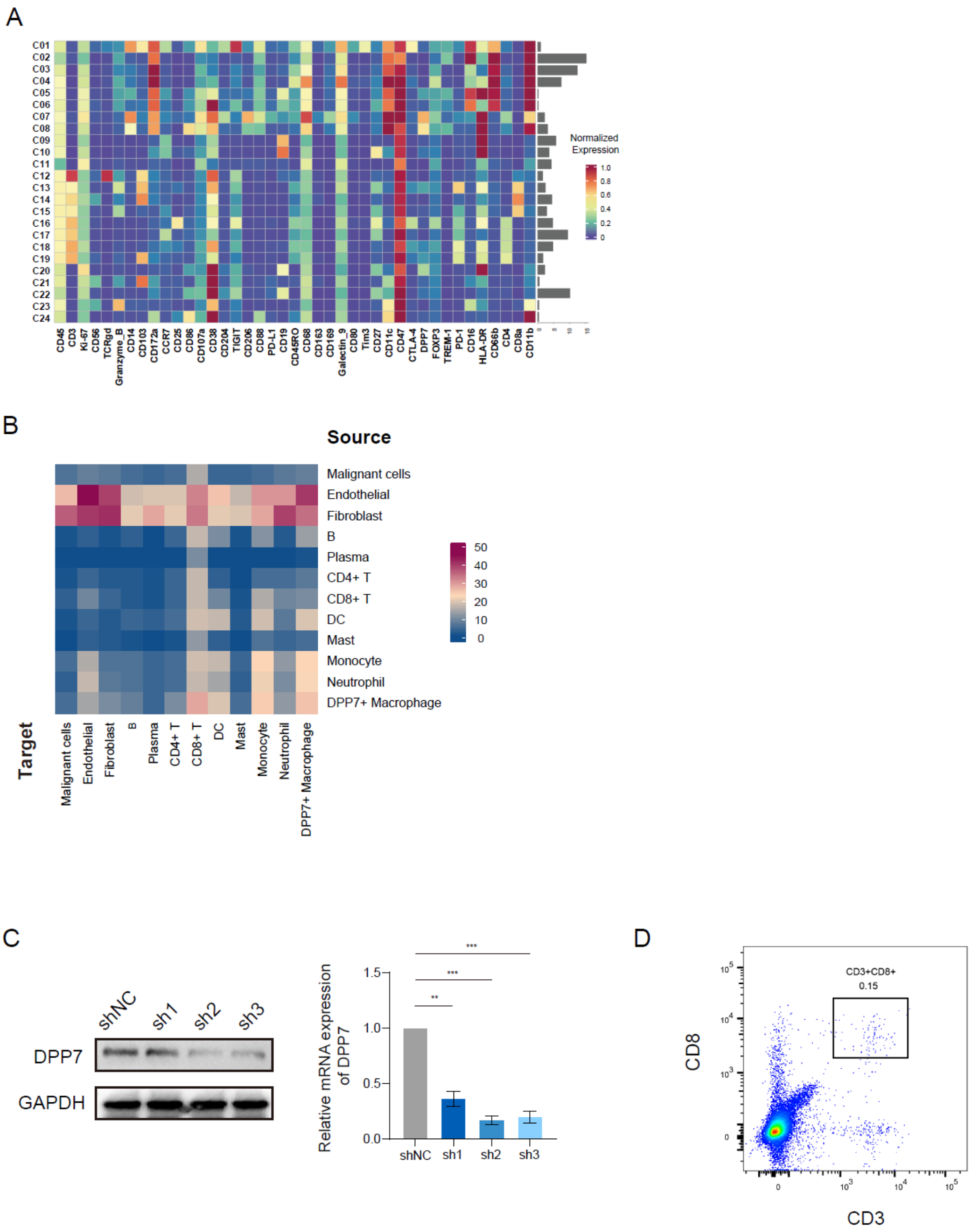

Supplementary Figure 4. DPP7⁺TAMs are associated with the immune exhausted contexture and exerted an immunosuppressive function in CRC. (A) Heatmap of all markers expression across all subclusters. (B) The cell-cell communication analysis based on scRNA-seq data. (C) The levels of protein and mRNA of TCM-educated shDPP7/shNC-transduced BMDMs. DPP7-sh2 demonstrates the most effective gene knockdown efficiency, thus we choose DPP7-sh2 as shDPP7-BMDMs to accomplish the following cell and animal experiments. (D) FCM validation of the efficiency of CD8⁺T

depletion. *p<0.05, **p<0.01, ***p<0.001, and ns = non-significant.

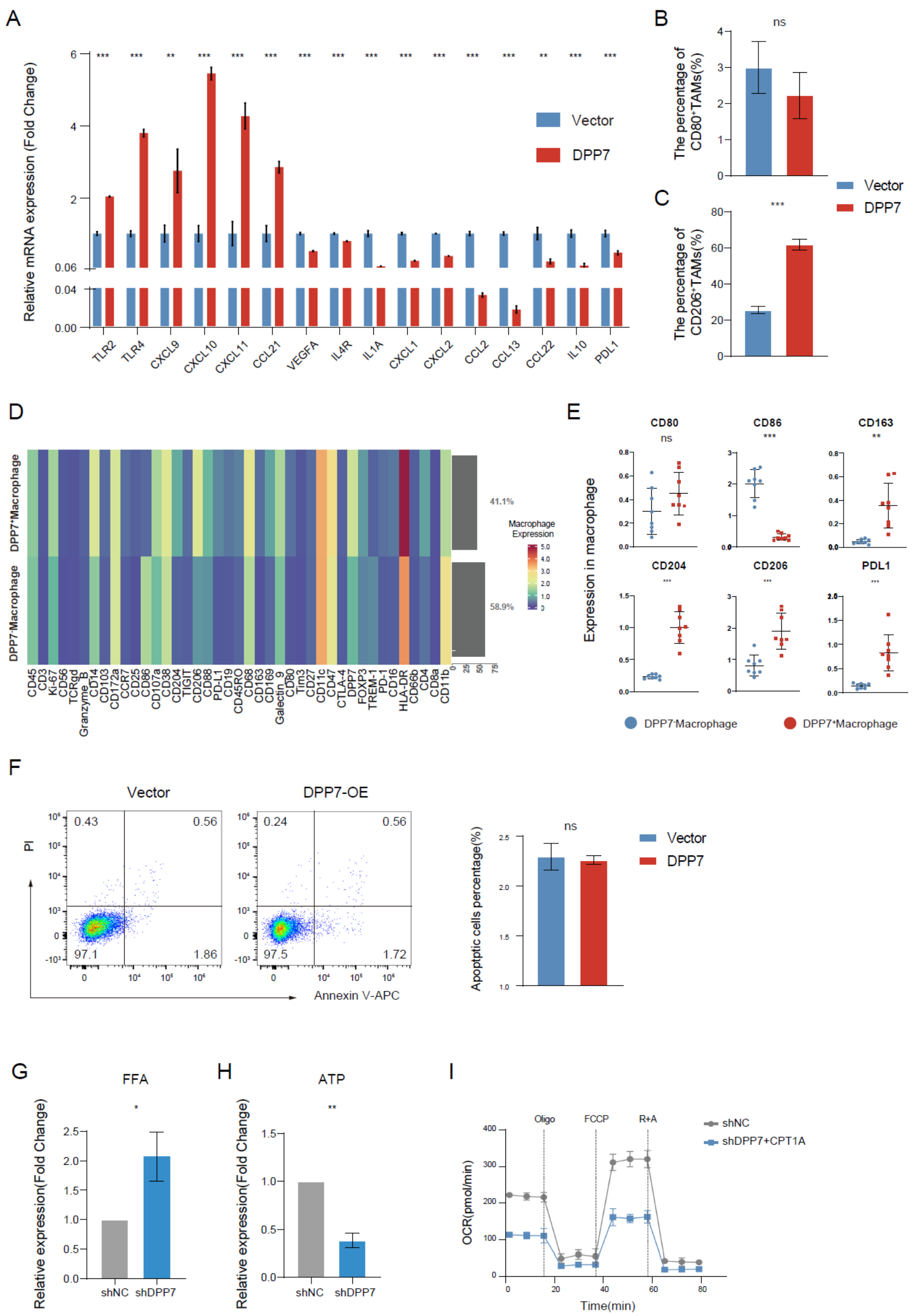

Supplementary Figure 5. Metabolic characterization caused by DPP7. (A) qPCR analyses of the indicated genes in Vector and DPP7 groups in THP-1-differentiated macrophages. (B) FCM results of CD80⁺TAMs percentage between Vector and DPP7 groups in THP-1-differentiated macrophages. (C) FCM results of CD206⁺TAMs

percentage between Vector and DPP7 groups in THP-1-differentiated macrophages. (D) The expression of all markers in macrophages between DPP7^{+}Macrophage and DPP7^{-}Macrophage groups based on cyTOF. (E) The expression of M1 and M2 markers in macrophages between DPP7^{+}Macrophage and DPP7^{-}Macrophage groups based on cyTOF. (F) FCM results of apoptotic cells percentage between Vector and DPP7 groups in THP-1-differentiated macrophages. (G) Intracellular FFAs content in TCM-educated shNC/shDPP7 BMDMs detected by assay kit. (H) Intracellular ATP content in TCM-educated shNC/shDPP7 BMDMs detected by assay kit. (I) The OCR was measured at baseline and in response to incubation with oligomycin (Oligo), FCCP (Carbonyl Cyanide 4-(trifluoromethoxy) phenylhydrazone), and rotenone plus antimycin A (R + A) in TCM-educated shNC/shDPP7 BMDMs. *p<0.05, **p<0.01, ***p<0.001, and ns = non-significant.

A

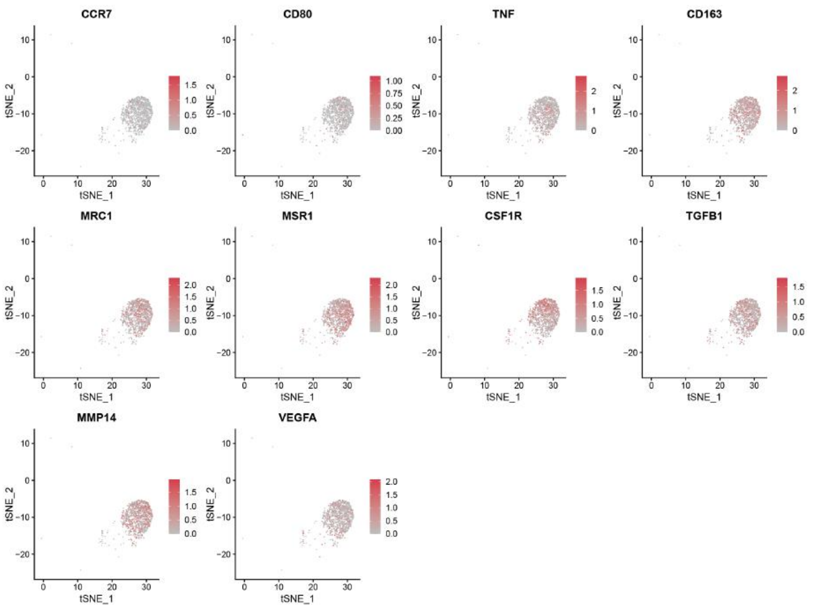

B

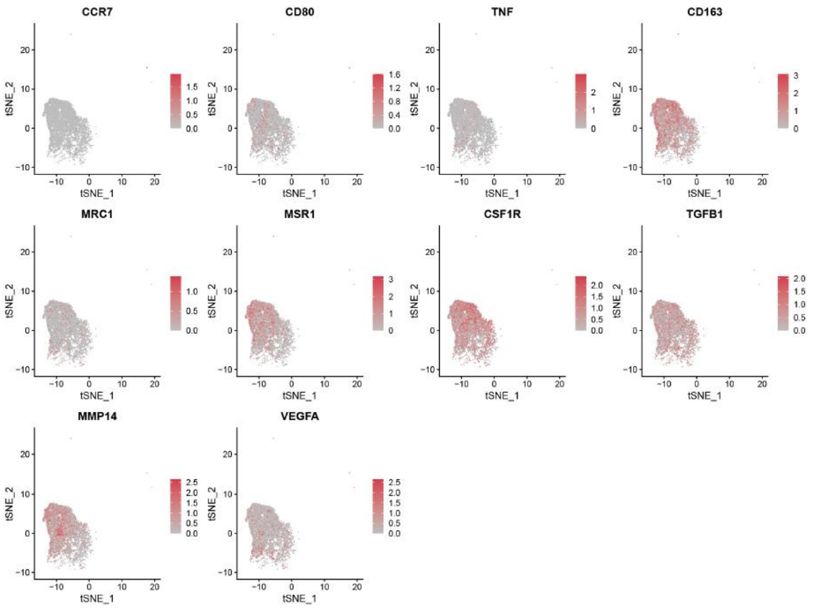

Supplementary Figure 6. The expression of M1/M2 polarization markers in DPP7^{+}TAM. (A) Classic markers of DPP7^{+}TAMs from scRNA-seq dataset (GSE178341). (B) Classic markers of DPP7^{+}TAMs from scRNA-seq dataset (PRJNA748525).

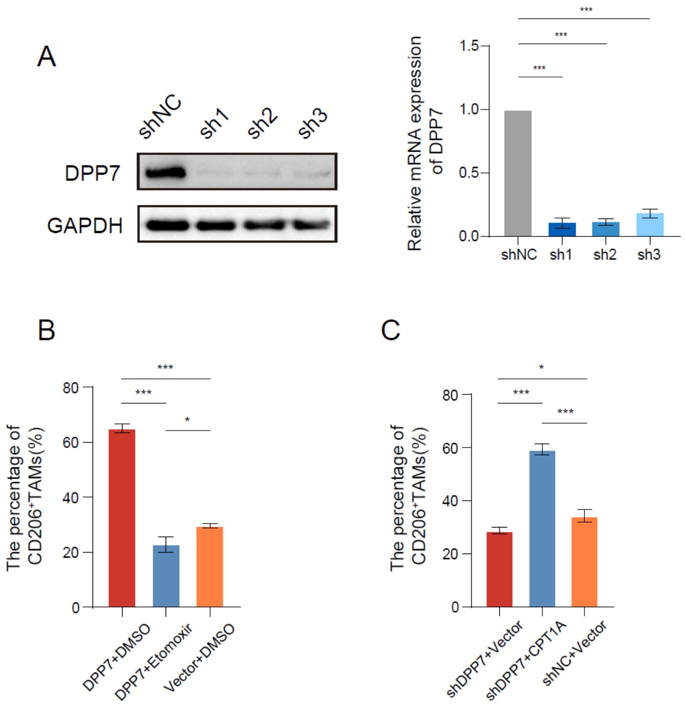

Supplementary Figure 7. DPP7 enhances FAO by interacting with CPT1A and reducing its ubiquitination-dependent degradation. (A) The levels of protein and mRNA of DPP7 in shDPP7/shNC-transduced THP-1-differentiated macrophages. DPP7-sh1 demonstrates the most effective gene knockdown efficiency, thus we choose DPP7-sh1 as shDPP7 in THP-1-differentiated macrophages to accomplish the relative experiments. (B) FCM results of $CD206^+$ percentage between DPP7+DMSO, DPP7+Etomoxir and Vector+DMSO groups in THP-1-differentiated macrophages. (C) FCM results of $CD206^+$ percentage between shDPP7+Vector, shDPP7+CPT1A and shNC+Vector groups in THP-1-differentiated macrophages. *$p<0.05$, **$p<0.01$, ***$p<0.001$, and ns = non-significant.

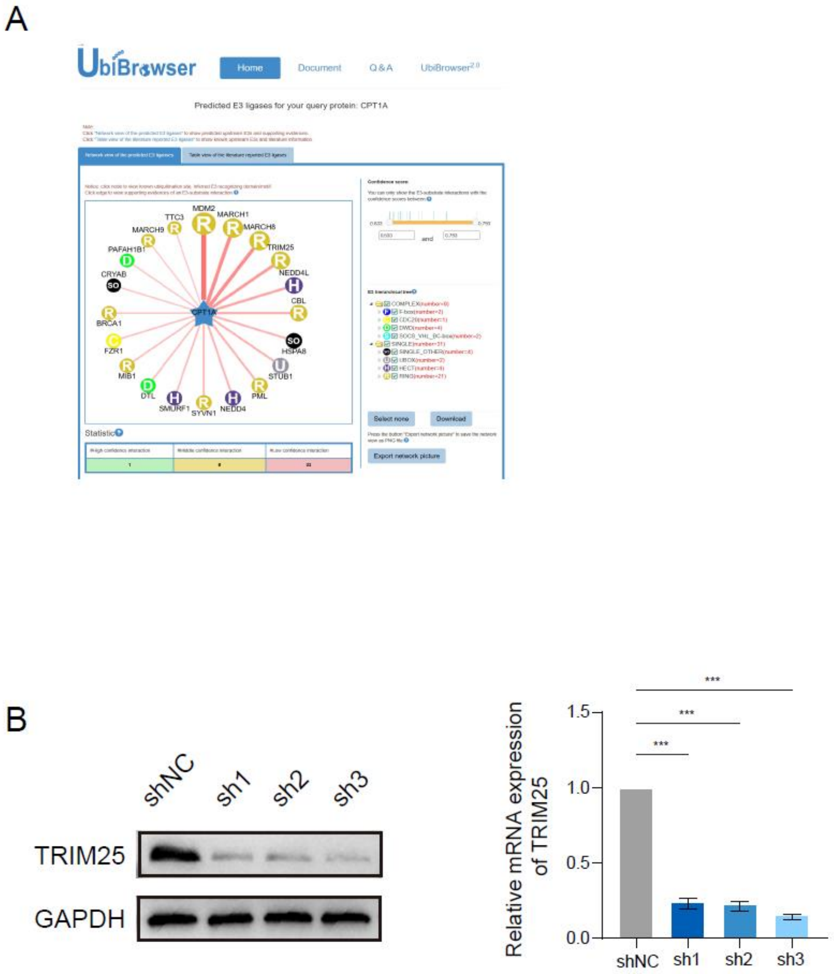

Supplementary Figure 8. DPP7 reduces the ubiquitination-induced degradation of CPT1A by interacting with CPT1A in a mutually exclusive manner with TRIM25. (A) The results of prediction about E3 ubiquitin ligase based on UbiBrowser website. (B) The levels of protein and mRNA of shTRIM25/shNC-transduced THP-1-differentiated macrophages. TRIM25-sh3 demonstrates the most effective gene knockdown efficiency, thus we choose TRIM25-sh3 as shTRIM25 in THP-1-differentiated macrophages to accomplish the following experiments. *p<0.05, **p<0.01, ***p<0.001, and ns = non-significant.

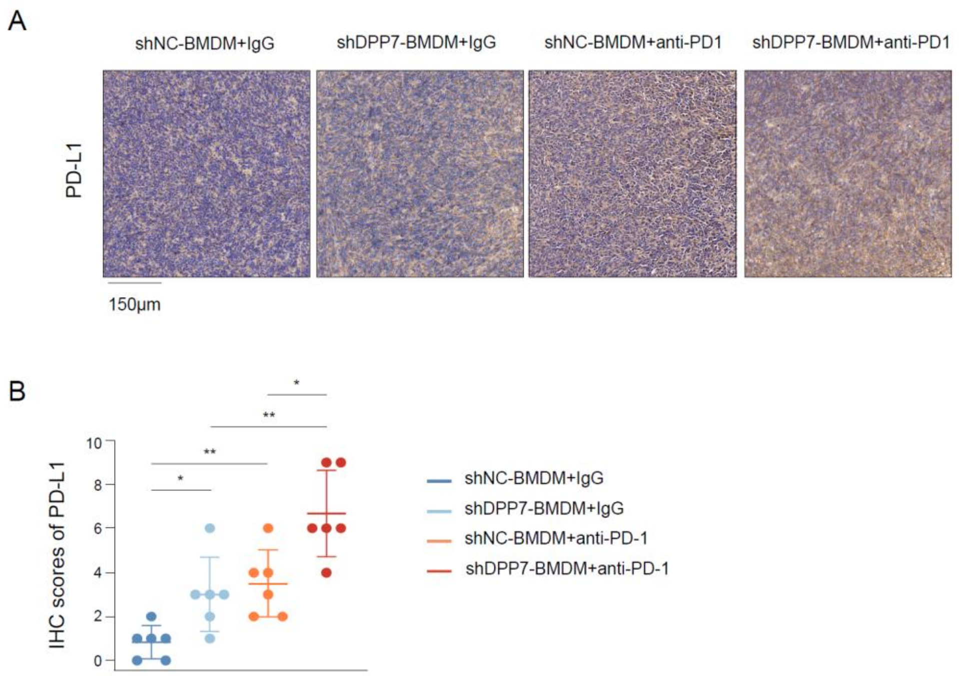

Supplementary Figure 9. DPP7 knockdown in BMDMs enhances the efficacy of PD-1 blockade and reverse the immunosuppressive TIME. (A) The expression of PD-L1 based on IHC in different treatment groups. (B) IHC scores of PD-L1 in different treatment groups.

Supplementary Table 1. Baseline of clinicopathological characteristics of Huadong cohort

	Primary cohort
**Factors**	**No (%)**
**All patients**	282(%)
**Gender**	
Female	119(42.2%)
Male	163(57.8%)
**Age**	
⩽60	37(13.1%)
＞60	245(86.9%)
**Preoperative serum CEA (ng/ml)**	
⩽5	154(54.6%)
＞5	128(45.4%)
**Tumor location**	
Left-sided colon	98(34.8%)
Right-sided colon	56(19.8%)
Rectum	128(45.4%)
**Tumor size (cm)**	
⩽4	166(58.9%)
＞4	116(41.1%)
**Histology**	
Non-adenocarcinoma	30(10.6%)
Adenocarcinoma	252(89.4%)
**Differentiation**	
Well/moderately	243(86.2%)
Poorly/undifferentiated	39(13.8%)
**T stage**	
T1-2	101(35.8%)
T3-4	181(64.2%)
**N stage**	
N0	145(51.4%)
N1-2	137(48.6%)
**M stage**	
M0	226(80.1%)
M1	56(19.9%)
**TNM stage**	
I	47(16.7%)
II	74(26.2%)
III	105(37.2%)
IV	56(19.9%)
**MMR status**	
pMMR	263(93.3%)

dMMR	19(6.7%)

CEA, carcinoembryonic antigen; MMR, mismatch repair.

Supplementary Table 2. Relationship between the density of DPP7^{+}TAMs and clinical characteristics

	DPP7^{+}TAMs		
	**Low (%)**	**High (%)**	**P**
**All patients**	144(51.1%)	138(48.9%)	
**Gender**			0.670
Female	59(41.0%)	60(43.5%)	
Male	85(59.0%)	78(56.5%)	
**Age**			0.753
≤60	18(12.5%)	19(13.8%)	
＞60	126(87.5%)	119(86.2%)	
**Preoperative serum CEA(ng/ml)**			**0.025**
≤5	88(61.1%)	66(47.8%)	
＞5	56(38.9%)	72(52.2%)	
**Tumor location**			0.838
Left-sided colon	51(35.4%)	47(34.1%)	
Right-sided colon	30(20.8%)	26(18.8%)	
Rectum	63(43.8%)	65(47.1%)	
**Tumor size(cm)**			**0.046**
≤4	93(64.6%)	73(52.9%)	
＞4	51(35.4%)	65(47.1%)	
**Histology**			0.516
Non-adenocarcinoma	17(11.8%)	13(9.4%)	
Adenocarcinoma	127(88.2%)	125(90.6%)	
**Differentiation**			0.472
Well/moderately	122(84.7%)	121(87.7%)	
Poorly/undifferentiated	22(15.3%)	17(12.3%)	
**T stage**			0.065
T1-2	59(41.0))	42(30.4%)	
T3-4	85(59.0%)	96(69.6%)	
**N stage**			0.481
N0	77(53.5%)	68(49.3%)	
N1-2	67(46.5%)	70(50.7%)	
**M stage**			**0.048**
M0	122(84.7%)	104(75.4%)	
M1	22(15.3%)	34(24.6%)	
**TNM stage**			0.192
I	28(19.4%)	19(13.8%)	
II	40(27.8%)	34(24.6%)	
III	54(37.5%)	51(37.0%)	
IV	22(15.3%)	34(24.6%)	
**MMR status**			0.537
pMMR	133(92.4%)	130(94.2%)	

dMMR	11(7.6%)	8(5.8%)

CEA, carcinoembryonic antigen; MMR, mismatch repair.

Supplementary Table 3. Relationship between DPP7 and CD68 expression in Huadong patient cohort based on IHC staining (n=282)

		DPP7			P value
		High	Low	Total	
CD68	High	50(57.5%)	78(40%)	128(45.4%)	0.007
	Low	37(42.5%)	117(60%)	154(54.6%)	
	Total	87(30.9%)	195(69.1%)	282(100.0%)	

Supplementary Table 4. Up-regulated proteins binding to DPP7 control to IgG, involving in fatty acid metabolism or fatty acid degradation pathways

No.	Protein	DPP7	IgG	FC
1	CPT1A	120400000	36237.5	3322.5
2	HADHB	70254000	36237.5	1938.7
3	ACSL4	17191000	36237.5	474.4
4	HSD17B12	11550000	36237.5	318.7
5	ELOVL1	9380000	36237.5	258.8
6	ACOX1	5299400	36237.5	146.2
7	ACADVL	5232800	36237.5	144.4
8	ECHS1	6989600	36237.5	137.7
9	ACAA2	3533300	36237.5	97.5
10	HSD17B4	27113000	469140	57.8
11	TECR	1049800	36237.5	29.0
12	ACOX3	633870	36237.5	17.49
13	ALDH2	192270	36237.5	5.3

Supplementary Table 5. Antibodies used in the CyTOF assay

Label	Marker	Clone	Manufacturer
89Y	CD45	HI30	BioLegend
115In	CD3	UCHT1	Bio Cell
139La	Ki-67	SolA15	eBioscience
141Pr	CD56	NCAM16.2	BD
142Nd	TCR γ/δ	5A6.E9	Thermo
143Nd	Granzyme B Recombinant	QA16A02	BioLegend
144Nd	CD14	M5E2	BioLegend
145Nd	CD103	B-Ly7	eBioscience
146Nd	CD172a(SIRPα)	15-414	BioLegend
147Sm	CD197(CCR7)	G043H7	BioLegend
149Sm	CD25(IL-2Rα)	24212	R&D
150Nd	CD86	Fun-1	BD
151Eu	CD107a(LAMP-1)	H4A3	BioLegend
152Sm	CD38	HIT2	BioLegend
153Eu	CD204(SR-AI)	351615	R&D
154Sm	TIGIT(VSTM3)	A15153G	BioLegend
155Gd	CD206(MMR)	15-2	BioLegend
156Gd	CD88(C5aR)	S5/1	BioLegend
157Gd	CD274(B7-H1,PD-L1)	29E.2A3	BioLegend
158Gd	CD19	HIB19	BioLegend
159Tb	CD45RO	UCHL1	BioLegend
160Gd	CD68	Y1/82A	BioLegend
161Dy	CD163	GHI/61	BioLegend
162Dy	CD169(Siglec-1)	7-239	BioLegend
163Dy	Galectin-9	9M1-3	BioLegend
164Dy	CD80(B7-1)	2D10.4	eBioscience

165Ho	CD366(Tim-3)	F38-2E2	BioLegend
167Er	CD27	O323	BioLegend
168Er	CD11c	BU15	BioLegend
169Tm	CD47	CC2C6	BioLegend
170Er	CD152(CTLA-4)	14D3	eBioscience
171Yb	DPP7	28-57	SCBT
172Yb	FOXP3	PCH101	eBioscience
173Yb	TREM-1	193015	R&D
174Yb	CD279(PD-1)	EH12.2H7	BioLegend
175Lu	CD16	3G8	BioLegend
176Yb	HLA-DR	L243	BioLegend
195Pt	CD66b	G10F5	BioLegend
197Au	CD4	RPA-T4	BioLegend
198Pt	CD8a	RPA-T8	BioLegend
209Bi	CD11b	M1/70	BioLegend

Supplementary Table 6. The sequences of the shRNA

Gene	Sequences
shDPP7(mice)-1	CCATTTCAACTTTGAGAGTTT
shDPP7(mice)-2	CCAGACTAAAGCAACCAGCAA
shDPP7(mice)-3	CCTGACTTTCATGAGAATTAT
shDPP7(human)-1	CCAGCAACAATGTGACCGATA
shDPP7(human)-2	CGAGCACTGCTACGACATCTA
shDPP7(human)-3	CCGACAGATCAAGGACTTGTT
shTRIM25(human)-1	GAGTGAGATCCAGACCTTGAA
shTRIM25(human)-2	GAACTGAACCACAAGCTGATA
shTRIM25(human)-3	CCGGAACAGTTAGTGGATTTA

Supplementary Table 7. The sequences of the primers

Gene	Primer Sequences
DPP7(mice)	Forward: ATGAACTTCCATCCTTGTTACCC<br>Reverse: TGAAATGGTCCATGTATTGCTCA
DPP7(human)	Forward: GGACCACTTCAACTTCGAGC<br>Reverse: GCCCTCGTTCCCAGTGTAG
CPT1A(human)	Forward: ATCAATCGGACTCTGGAAACGG<br>Reverse: TCAGGGAGTAGCGCATGGT
TRIM25(human)	Forward: AATCGGCTGCGGGAATTTTTC<br>Reverse: TCTCACATCATCCAGTGCTCT
GAPDH(human)	Forward: GGAGCGAGATCCCTCCAAAAT<br>Reverse: GGCTGTTGTCATACTTCTCATGG
IL-1A(human)	Forward: TGGTAGTAGCAACCAACGGGA<br>Reverse: ACTTTGATTGAGGGCGTCATTC
IL-4R(human)	Forward: CGTGGTCAGTGCGGATAACTA<br>Reverse: TGGTGTGAACTGTCAGGTTTC
IL-10(human)	Forward: GACTTTAAGGGTTACCTGGGTTG<br>Reverse: TCACATGCGCCTTGATGTCTG
TLR2(human)	Forward: ATCCTCCAATCAGGCTTCTCT<br>Reverse: GGACAGGTCAAGGCTTTTTACA
TLR4(human)	Forward: AGACCTGTCCCTGAACCCTAT<br>Reverse: CGATGGACTTCTAAACCAGCCA
VEGFA(human)	Forward: AGGGCAGAATCATCACGAAGT<br>Reverse: AGGGTCTCGATTGGATGGCA
CD274(PD-L1, human)	Forward: TGGCATTTGCTGAACGCATTT<br>Reverse: TGCAGCCAGGTCTAATTGTTTT
CXCL1(human)	Forward: ATGGCCCGCGCTGCTCTC<br>Reverse: CCCTTCTGGTCAGTTGGATTTG
CXCL2(human)	Forward: GGGAATTCACCTCAAGAACATC

	Reverse: GACAAGCTTTCTGCCCATTC
CXCL9(human)	Forward: CCAGTAGTGAGAAAGGGTCGC
	Reverse: AGGGCTTGGGGCAAATTGTT
CXCL10(human)	Forward: GTGGCATTCAAGGAGTACCTC
	Reverse: TGATGGCCTTCGATTCTGGATT
CXCL11(human)	Forward: GACGCTGTCTTTGCATAGGC
	Reverse: GGATTTAGGCATCGTTGTCCTTT
CCL2(human)	Forward: GCTCATAGCAGCCACCTCATTC
	Reverse: CCGCCAAAATAACCGATGTGATAC
CCL13(human)	Forward: CTCAACGTCCCATCTACTTGC
	Reverse: TCTTCAGGGTGTGAGCTTTCC
CCL21(human)	Forward: GTTGCCTCAAGTACAGCCAAA
	Reverse: AGAACAGGATAGCTGGGATGG
CCL22(human)	Forward: ATCGCCTACAGACTGCACTC
	Reverse: GACGGTAACGGACGTAATCAC

Supplementary Table 8. Antibodies used in WB and Co-IP

Antibody	Concentration	Application	Cat #	Company
DPP7	1:1000	WB	19018-1-AP	Proteintech
CPT1A	1:5000	WB	15184-1-AP	Proteintech
TRIM25	1:1000	WB	12573-1-AP	Proteintech
GAPDH	1:50000	WB	60004-1-Ig	Proteintech
Myc	1:2000	WB	60003-2-Ig	Proteintech
Flag	1:20000	WB	20543-1-AP	Proteintech
GST	1:5000	WB	66001-2-Ig	Proteintech
His	1:5000	WB	66005-1-Ig	Proteintech
ubiquitin	1:1000	WB	10201-2-AP	Proteintech
HRP-conjugated Goat Anti-Mouse IgG	1:5000	WB	SA00001-1	Proteintech
HRP-conjugated Goat Anti-Rabbit IgG	1:5000	WB	SA00001-2	Proteintech
DPP7	25 μg/mL	Co-IP	PA5-47489	Thermo
CPT1A	1:30	Co-IP	ab220789	Abcam

Supplementary Table 9. Antibodies used in FCM

Antibody	Cat #	Company
FITC anti-human CD206 (MMR)	321104	BioLegend
PE anti-human CD80	375410	BioLegend
anti-mouse CD45 BV510	103137	BioLegend
fixable viability dye NIR	65-0865-14	Thermo
anti-mouse CD3 APC	17-0031-82	Thermo
anti-mouse CD3 PerCP/Cyanine5.5	100218	BioLegend
anti-mouse CD8 FITC	11-0081-85	Thermo
anti-mouse CD279 BV421(PD1)	135218	BioLegend
anti-mouse CD279 BV650 (PD1)	135243	BioLegend
anti-mouse Granzyme B Alexa Fluor® 700	372222	BioLegend
anti-mouse IFN-γ BV605	505840	BioLegend
anti-mouse CD11b BUV395	363-0112-82	Thermo
anti-mouse F480 BV421	123137	BioLegend
anti-mouse CD80 APC	17-0801-82	Thermo
anti-mouse MHC-II FITC	11-5321-82	Thermo
anti-mouse CD206 PE	141706	BioLegend
